# Genome-Wide Association Study on Three Behaviors Tested in an Open Field in Heterogeneous Stock Rats Identifies Multiple Loci Implicated in Psychiatric Disorders

**DOI:** 10.3389/fpsyt.2022.790566

**Published:** 2022-02-14

**Authors:** Mustafa Hakan Gunturkun, Tengfei Wang, Apurva S. Chitre, Angel Garcia Martinez, Katie Holl, Celine St. Pierre, Hannah Bimschleger, Jianjun Gao, Riyan Cheng, Oksana Polesskaya, Leah C. Solberg Woods, Abraham A. Palmer, Hao Chen

**Affiliations:** ^1^Department of Pharmacology, Addiction Science and Toxicology, University of Tennessee Health Science Center, Memphis, TN, United States; ^2^Department of Psychiatry, University of California, San Diego, La Jolla, CA, United States; ^3^Department of Internal Medicine, Wake Forest School of Medicine, Winston Salem, NC, United States; ^4^Institute for Genomic Medicine, University of California, San Diego, La Jolla, CA, United States

**Keywords:** GWAS, outbred, anxiety, open field, novelty-seeking, social interaction, heterogeneous stock, rats

## Abstract

Many personality traits are influenced by genetic factors. Rodents models provide an efficient system for analyzing genetic contribution to these traits. Using 1,246 adolescent heterogeneous stock (HS) male and female rats, we conducted a genome-wide association study (GWAS) of behaviors measured in an open field, including locomotion, novel object interaction, and social interaction. We identified 30 genome-wide significant quantitative trait loci (QTL). Using multiple criteria, including the presence of high impact genomic variants and co-localization of cis-eQTL, we identified 17 candidate genes (*Adarb2, Ankrd26, Cacna1c, Cacng4, Clock, Ctu2, Cyp26b1, Dnah9, Gda, Grxcr1, Eva1a, Fam114a1, Kcnj9, Mlf2, Rab27b, Sec11a, and Ube2h*) for these traits. Many of these genes have been implicated by human GWAS of various psychiatric or drug abuse related traits. In addition, there are other candidate genes that likely represent novel findings that can be the catalyst for future molecular and genetic insights into human psychiatric diseases. Together, these findings provide strong support for the use of the HS population to study psychiatric disorders.

## 1. Introduction

Many personality traits are predictors of vulnerability to addiction (1). For example, individuals with symptoms of anxiety are more likely to be smokers (2, 3), and novelty seeking is positively correlated with both smoking onset (4) and cocaine abuse (5). In addition, the social environment plays a critical role in the development and treatment of addiction (6). Many of these phenomena can be modeled using rodents to unveil their neural, genetic, and molecular mechanisms (7–10).

The open-field test (OFT) is a widely used behavioral test for measuring anxiety-like and exploratory behavior in rodents (11–14). A rodent is typically placed in an open chamber surrounded by tall walls. Video recording of the rodent's locomotor movements is then analyzed. In general, rats spend most of the testing session walking along the wall (i.e., thigmotaxis). Increased time spent in the center of the area or decreased latency to enter the center are interpreted as indications of lower anxiety. The OFT is widely used to model anxiety and is sensitive to the anxiolytic-like effects of classical benzodiazepines, and 5-HT1A receptor agonists (11). The novel object interaction test (NOIT) is usually conducted in an open arena where a novel object is placed in the center. The time spent and distance traveled around the object zone are used as indicators of preference for novelty. Novel object interaction has been considered as an important predictor in addiction-like traits (15, 16) and high novelty preference increases the propensity for addictive drug-seeking behavior (9, 17, 18). There are multiple different methods for conducting social interaction test (SIT) in rats (19–21). In general, an unfamiliar stimulus rat and the rats to be tested are placed in the same arena. While manual scoring of social interaction often allows both rats to be freely moving, experiments using automated video analysis often limit the movement of the stimulus rat. Computer algorithm then extract the time spend and distance traveled by the test rat around the stimulus rat, which reflects the social tendency of the test rat.

The heterogeneous stock (HS) rats were originally derived from interbreeding eight inbred strains (22). An analysis on these founders reported 7.2 million single nucleotide variants (23). This population has been maintained as outbred for more than 90 generations. The chromosomes of individuals in this population represent a genetic mosaic of the founders' haplotypes, with the average distance between recombination events in the centiMorgan range (24). This allows for genetic mapping to only a few million bases (Mb), a much smaller region than what can be identified using traditional F2 intercross or backcross mapping strategies. Several high-resolution genome-wide association studies (GWAS) (23, 25–27) have been successfully carried out. Here we report the results on associations of genomic loci with measures obtained from OFT, NOIT and SIT. These analyses were based on an expanded data set that contained about twice the sample size of that reported previously (28). These data were collected as part of a larger GWAS on socially acquired nicotine intravenous self-administration, which will be the subject of a separate publication.

## 2. Materials and Methods

### 2.1. Animals

The N/NIH heterogeneous stock (HS) rat (RRID:RGD_2314009), was created at the NIH in 1984 by interbreeding the following eight inbred founder strains: ACI/N, BN/SsN, BUF/N, F344/N, M520/N, MR/N, WKY/N and WN/N (22). The HS rats used in this study were sent from The Medical College of Wisconsin to the University of Tennessee Health Science Center (UTHSC) at 3–6 weeks of age. A total of 16 batches of HS rats were transferred between October 27, 2014 and September 20, 2018. Each batch consisted of 25 males and 25 females that were used as breeders. After a 2-week quarantine period, rats were transferred to a reversed 12 h light-dark cycle (lights off at 9:00 a.m.) housing room. Breeding pairs were assigned according to an algorithm that maximized the genetic diversity of the offspring. Litters were culled to a maximum of 8 pups to ensure a consistent nutritional environment. Rats were weaned on postnatal day (PND) 21. A radio frequency identification (RFID) chip was inserted subcutaneously into each rat at the time of weaning. Two male and two female rats per litter were used for behavioral studies. Sprague-Dawley (SD) rats (20 for each sex, purchased from Harlan Laboratories, Madison, WI, RRID:RGD_737903) were used as the stimulus rats in the social interaction test. Teklad Irradiated LM-485 Mouse/Rat Diet and water were provided *ad libitum*. All rats were group-housed with 2–4 same-sex peers throughout the experiments to avoid social isolation. All procedures were conducted in accordance with the NIH Guidelines concerning the Care and Use of Laboratory Animals, as approved by the Institutional Animal Care and Use Committee of the University of Tennessee Health Science Center.

### 2.2. Study Design

All HS rats (626 males and 620 females in total from 16 batches) were adolescents when tests began. Their age was 31.8 ± 2.6 (mean ± STD) on the day of the OFT. Adolescent rats were used because the onset of many psychiatric diseases occur during this age (29). Each HS rat was tested in all three behavioral tests, one test per day, in the following sequence: OFT, NOIT, and SIT. All tests were conducted in the dark phase of the light cycle (9 a.m.–4 p.m.) and were conducted in the same open field and recorded using the same video capture system.

### 2.3. Behavioral Testing Procedure

#### 2.3.1. Open Field Test

Two OFT arenas were constructed using black acrylic glass, measuring 100 cm (L) × 100 cm (W) × 50 cm (H), which were placed side by side. The floors were covered by wood boards painted with either black or white acrylic paint (ART-Alternatives, ASTM D-4236, Emeryville, CA, USA) to contrast the coat of the animals (i.e., a black board was used for rats with white fur). The test chambers were illuminated by a long-range, 850-nm infrared light (LIR850-70, LDP LLC, Carlstadt, NJ) located 160 cm above the center of the two test chambers. No source of visible light was present during behavioral testing, with the exception of a flat panel monitor (Dell 1908FP). A digital camera (Panasonic WV-BP334) fitted with an 830 nm infrared filter (X-Nite830-M37, LTP LLC, Carlstadt, NJ) and located next to the infrared light source was used to record the behavior of the rats. All rats were released at the same corner of the test chamber, and data were collected for 1 h.

#### 2.3.2. Novel Object Interaction Test

This test was conducted the day after the OFT in the same arena. A cylindrical rat cage constructed using 24 aluminum rods (30 cm in length) spaced 1.7 cm apart was used as the novel object. The bottom and top of the cage (15 cm in diameter) were manufactured using a 3D printer from polylactic acid. The design can be downloaded from https://github.com/chen42/RatSocialInteractionTest. The novel object was placed in the center of the arena before testing. The test duration was 20 min and was recorded using the same camera as that used in the OFT.

#### 2.3.3. Social Interaction Test

This test was conducted the day after the NOIT. This test compares the preference of a subject rat for a stimulus rat restricted in a cylindrical cage (i.e., the novel object used in the NOIT) against an empty cylindrical cage. The test arena was reduced to 100*cm*(*L*) × 60*cm*(*W*) × 50*cm*(*H*) by using a black board placed vertically in the arena. Two cylindrical cages described above were placed ~30 cm away from the walls on opposite sides (i.e., similar to the arrangement commonly used in the three-chamber test). A randomly selected stimulus Sprague-Dawley rat of the same sex and similar weight as the HS test rat was placed into one of the cylindrical cages (kept the same throughout the experiment) 5 min before the HS subject rat was placed into the arena. The stimulus and subject rats were never housed together and thus were unfamiliar to each other. No social isolation was conducted on either rat. Each stimulus rat was used no more than once per day. The test duration was 20 min and was recorded using the same camera as that used in the OFT.

#### 2.3.4. Analysis of Video Data

Ethovision XT video tracking system (RRID:SCR_000441, Version 4.0, Noldus Information Technology, The Netherlands) was used to analyze the videos recorded in all behavioral tests. After identifying the arena and calibrating the size of the arena, specific zones in the arena were outlined. For OFT and NOIT, one center zone, which was a circular region with a diameter of 20 cm, was used. For the SIT, one object zone and one social zone, both were circular regions with diameters of 20 cm, corresponding to the two cylindrical cages, respectively, were specified. The extracted data included the total distance traveled in the arena, the duration and the frequency the test rat was present in specific zones, the distance of the subject to the zones, and the latency of the test rat entering the zones. The center of the subject rat was used for all calculations. Phenotypic correlations were determined using the Pearson test.

### 2.4. Pre-processing of Phenotype Data

All phenotype data were stored in the C-GORD (RRID:SCR_021866) relational database. For genetic analysis, each trait was quantile-normalized separately for males and females; this approach is similar to using sex as a covariate. Other relevant covariates (including age, batch number, and coat color) were identified for each trait, and covariate effects were regressed out if they were significant and if they explained more than 2% of the variance. Residuals were then quantile-normalized again, after which the data for each sex were pooled prior to further analysis. This approach removed mean differences due to sex; further, it did not attempt to model gene-by-sex interactions.

### 2.5. Genotyping and Estimates of Heritability

Genotypes were determined using genotyping-by-sequencing (GBS), as described previously (30). This produced approximately 3.5 million single nucleotide polymorphisms (SNP) with an estimated error rate <1%. Variants for X- and Y-chromosomes were not called. We used this set of SNPs for GWAS, genetic correlations, and heritability estimates. We used GCTA-GREML (31) analysis to estimate proportion of variance attributable to SNPs.

### 2.6. Genetic Mapping

GWAS analysis employed a linear mixed model, as implemented in the software GCTA (32), using a genetic relatedness matrix (GRM) to account for the complex family relationships within the HS population and the Leave One Chromosome Out (LOCO) method to avoid proximal contamination (33, 34). Significance thresholds were calculated using permutation. Because all traits were quantile normalized, we used the same threshold for all traits (35). To identify quantitative trait loci (QTL), we scanned each chromosome to determine if there was at least one SNP that exceeded the permutation-derived threshold of −*log*_10_(*p*) > 5.6, which was supported by a second SNP within 0.5 Mb that had a *p*-value that was within 2 − *log*_10_(*p*) units of the most significant SNP.

There could be more than one QTL on the same chromosome for one trait. We resolve the dependency and determine their locations as follows: we used the top SNP from the most significant QTL as a covariate and performed a second GWAS of the chromsome in question. If the resulting GWAS had an additional SNP with a *p*-value that exceeded our permutation-derived threshold, it was considered to be a second, independent locus. This process was repeated (including all previously significant SNPs as covariates), until no more QTLs were detected on a given chromosome. Linkage disequilibrium (LD) intervals for the identified QTL were determined by identifying those markers that had a high correlation coefficient with the peak marker (*r*^2^ = 0.6).

Genetic fine mapping were conducted using Credible Set analysis (36) and SuSieR (37). The analysis determines the 99% credible set by a Bayesian approach, that is the smallest set of SNPs in a genomic region that were 99% likely, to contain the causal SNPs. SuSieR also uses a Bayesian approach but also quantify uncertainty in which variants should be selected when multiple, highly correlated variants compete with one another.

We used fastENLoc (38) and a LD cutoff-based method to colocalize behavioral and gene expression QTLs. For the LD cutoff-based method, we retained those behavioral and gene expression QTLs where the top SNPs were in strong LD (i.e., *r*^2^ > 0.6). The gene expression data were collected from 88 naive adult HS rats. Five brain regions (prelimbic, infralimbic, and orbitofrontal cortex, lateral habenula, and nucleus accumbens core) were collected for RNA-seq from each rat (39).

## 3. Results

### 3.1. Sex Differences

We found that many of the traits measured in OFT, NOIT, and SIT are different between males and females (Supplementary Table S1). In OFT, with the exception of latency of entering the center zone, all traits have statistically significant sex differences. In addition, four out of six traits in NOIT and seven out of eleven traits in SIT are different between males and females. The range of effect size (Cohen's d) for statistically significant differences is (0.14, 0.31). Our genetic analysis quantile-normalized each trait separately for males and females. This approach removed mean differences due to sex and allowed us to combine males and females in the same analysis to increase the power of GWAS.

### 3.2. Phenotypic Correlations

**Figure 1:**
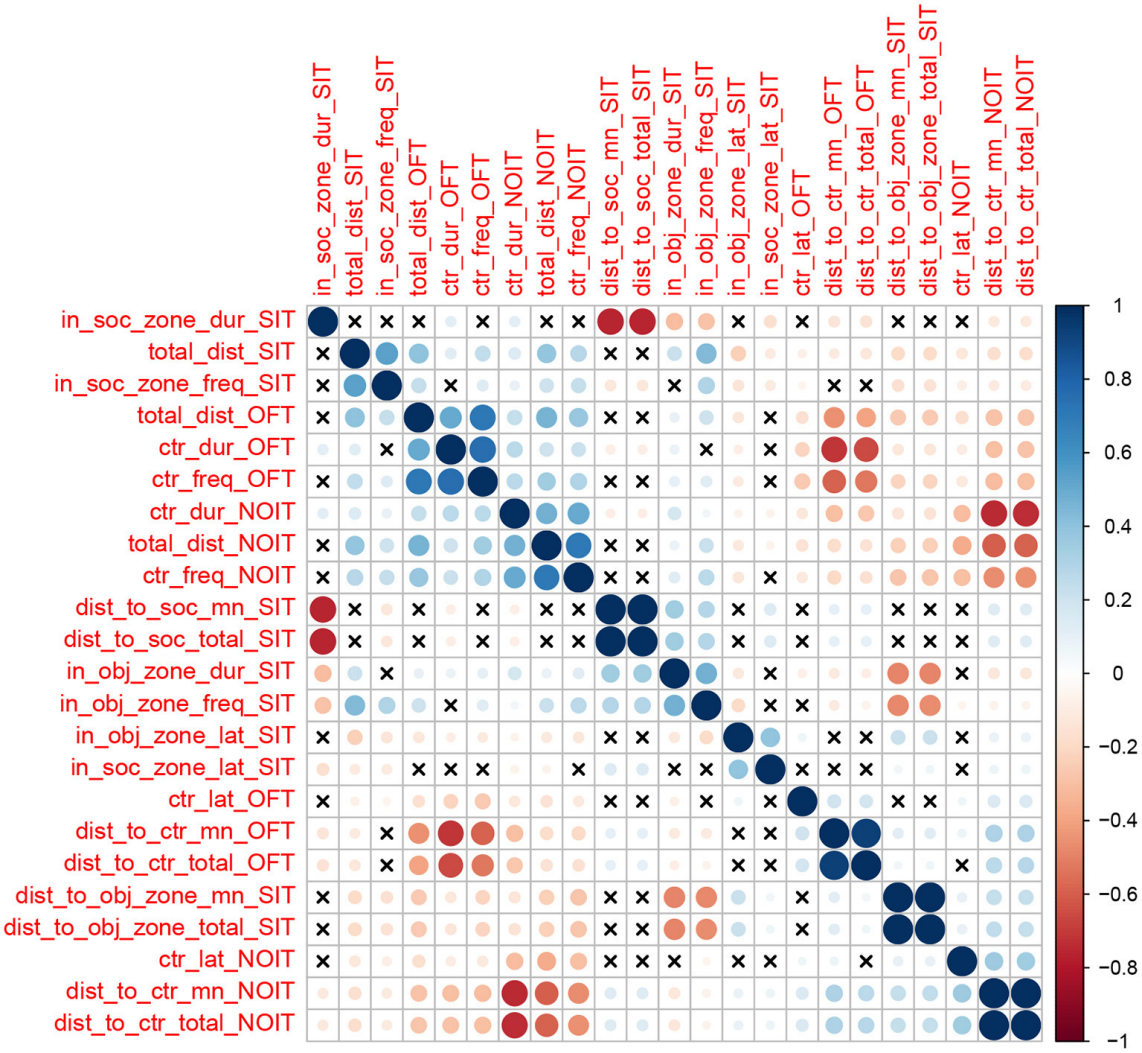
Heatmap showing the correlations between behavioral traits. The color scheme represents the direction of the correlation, whereas the intensity of the colors and the size of the circles are proportional to coefficients of the correlation. The cross signs indicates that the correlation of the two traits is not statistically significant (*p* > 0.05).

We calculated Pearson correlation between the 23 traits (Figure 1). We found 197 correlations with un-adjusted *p*-values < 0.05. Most of these correlations have relatively low Person coefficient (mean is 0.23, median is 0.18). However, due to the large sample size, most of these correlations are highly significant (median −*log*_10_(*p*) is 7.8). In general, correlations of traits obtained from the same behavioral test are among the strongest. For example, frequency of visiting the center and duration of staying in the center are positively correlated in OFT (*r* = 0.76), and duration in the social zone and distance to the social zone in the SIT are negatively correlated (*r* = –0.76). Most of these correlations are expected from the definitions of these variables.

**Figure 2:**
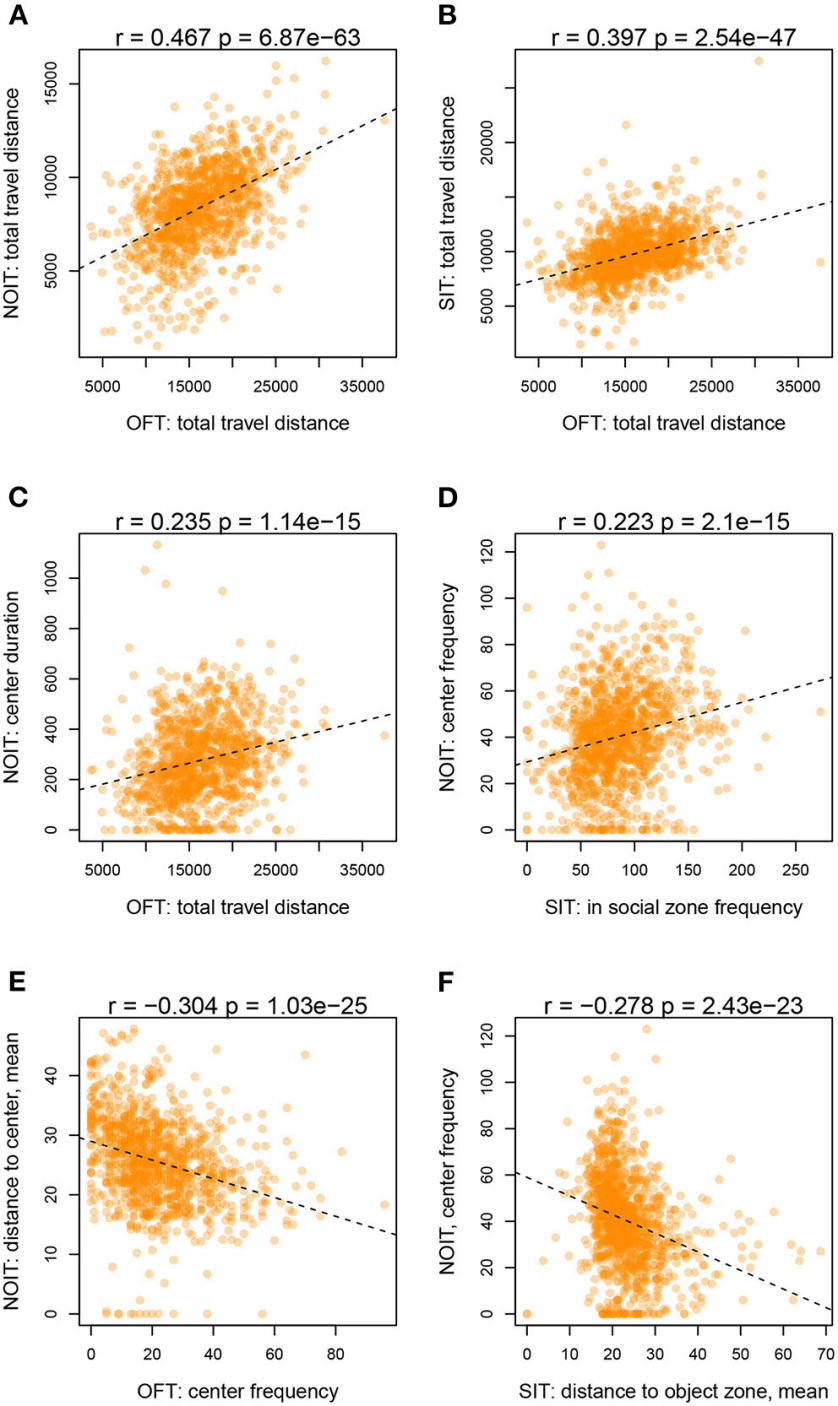
Selected scatter plots for correlation between behavioral tests shown in Figure 1. **(A)** OFT vs. NOIT. **(B)** OFT vs. SIT. **(C)** OFT vs. NOIT. **(D)** SIT vs. NOIT. **(E)** OFT vs. NOIT. **(F)** SIT vs. NOIT.

Among the correlations of variables derived from two different behavioral tests, correlations for measures of distance traveled are among the highest (range of Pearson r: 0.39–0.47, e.g., Figures 2A,B). Distance traveled in the OFT is also correlated with duration of center time in the NOIT (e.g., Figure 2C). Interestingly, the frequencies of visiting the center of the area in the NOIT is correlated with the frequency of visiting the social zone in the SIT (Figure 2D). In contrast, OFT center frequency is negatively correlated with NOIT mean distance to center in NOIT (Figure 2E), and distance to object zone in SIT is negatively correlated with center frequency in NOIT (Figure 2F).

### 3.3. Heritability

SNP heritability estimates (*h*^2^) for traits are provided in Table 1. In all the three behavioral tests, total travel distance has the highest heritability. In OFT, all heritability estimates are between 0.28 and 0.38, with the exception of that for latency of entering the center zone (*h*^2^ = 0.08). Heritability estimates for variables from the NOIT are slightly lower than that of the OFT; most of them are in the range of 0.21–0.29, with the exception of that for the latency of entering the center zone (*h*^2^ = 0.10). Heritability estimates for various measures of the SIT are in the range of 0.10–0.28. Interestingly, heritability estimates for measures on the social zone are consistently greater than those for the object zone.

**Table 1:** Heritability of open field (OFT), novel object (NOIT) and social interaction (SIT) tests.

**Test**	**Trait**	**Heritability ±SE**
OFT	Duration in center zone	0.284 ± 0.045
	Frequency of entering center zone	0.323 ± 0.044
	Latency of entering center zone	0.083 ± 0.034
	Mean distance to center zone	0.295 ± 0.043
	Total distance to center zone	0.300 ± 0.043
	Total travel distance	0.379 ± 0.044
NOIT	Duration in center zone	0.247 ± 0.043
	Frequency of entering center zone	0.209 ± 0.041
	Latency of entering center zone	0.100 ± 0.034
	Mean distance to center zone	0.249 ± 0.042
	Total distance to center zone	0.221 ± 0.041
	Total travel distance	0.287 ± 0.044
SIT	Duration in object zone	0.161 ± 0.037
	Duration in social zone	0.275 ± 0.040
	Frequency of entering object zone	0.177 ± 0.036
	Frequency of entering social zone	0.215 ± 0.036
	Latency of entering object zone	0.082 ± 0.032
	Latency of entering social zone	0.142 ± 0.034
	Mean distance to object zone	0.165 ± 0.038
	Mean distance to social zone	0.265 ± 0.041
	Total distance to object zone	0.153 ± 0.037
	Total distance to social zone	0.265 ± 0.041
	Total travel distance	0.281 ± 0.040

### 3.4. Identification of Multiple QTLs

In Table 2, we present SNPs that are significantly associated with the phenotypes. The genome-wide statistical significance of the association is determined by −*log*_10_*P* values greater than 5.609. For OFT, there are 9 significant loci for 5 traits. We did not find a significant QTL for *Duration in center zone* (*h*^2^ = 0.284±0.045). We identified two loci for *Frequency of entering center zone* and *Total travel distance*, 3 loci for *Total distance to center zone*. We found 4 NOIT traits have significant loci. Among them, *Total distance to center zone* has 3 loci and *Mean distance to center zone* has 2 loci. We did not find any significant loci for *Frequency of entering center zone* (*h*^2^ = 0.209 ± 0.041) and *Latency of entering center zone* (*h*^2^ = 0.100 ± 0.034). For SIT, we identified significant loci for all traits except *Latency of entering object zone* which has heritability of *h*^2^ = 0.082 ± 0.032. We found 2 loci for the traits *Latency of entering social zone, Mean distance to social zone Total distance to social zone* and *Total travel distance*. All genome-wide significant loci are shown in Figure 3. Genetic mapping of individual traits are shown as Manhattan plots as Supplementary Figures S1–S23. Regional association plots for representative traits are shown in Figures 4–6 for OFT, NOIT, and SIT, respectively. Other regional association plots are provided as Supplementary Figures S24–S50.

**Table 2:** QTL for open field (OFT), novel object interaction (NOIT), and social interaction (SIT) tests.

**Test**	**Trait**	**Top SNP**	**−*log*_10_*P***	**Interval size**	**Number of genes**
OFT	Frequency of entering center zone	chr1:24043699	5.714	0.12 Mb	5
	Frequency of entering center zone	chr4:118013062	5.777	2.0 Mb	47
	Latency of entering center zone	chr8:120910798	5.609	1.0 Mb	12
	Mean distance to center zone	chr4:58009499	7.469	2.4 Mb	60
	Total distance to center zone	chr4:58009499	7.254	2.4 Mb	60
	Total distance to center zone	chr4:118013062	6.099	2.0 Mb	47
	Total distance to center zone	chr14:44904830	5.741	2.1 Mb	44
	Total travel distance	chr10:94549701	7.286	4.2 Mb	98
	Total travel distance	chr11:33359859	8.268	0.92 Mb	23
NOIT	Duration in center zone	chr4:112234344	6.028	1.2 Mb	8
	Mean distance to center zone	chr4:112234344	6.598	1.2 Mb	8
	Mean distance to center zone	chr6:119975012	5.692	0.95 Mb	3
	Total distance to center zone	chr1:144080083	5.969	4.1 Mb	109
	Total distance to center zone	chr4:112234344	5.975	1.2 Mb	8
	Total distance to center zone	chr4:156801420	5.622	4.4 Mb	127
	Total travel distance	chr6:120117521	5.640	0.95 Mb	3
SIT	Duration in object zone	chr18:65869186	6.414	3.4 Mb	22
	Duration in social zone	chr4:151128675	5.820	2.9 Mb	34
	Frequency of entering object zone	chr13:90335374	5.827	1.1 Mb	46
	Frequency of entering social zone	chr1:239076581	7.273	0.27 Mb	6
	Latency of entering social zone	chr10:52831274	6.052	0.34 Mb	2
	Latency of entering social zone	chr17:58611795	6.104	0.86 Mb	1
	Mean distance to object zone	chr19:20666789	6.746	1.6 Mb	23
	Mean distance to social zone	chr19:55339863	6.661	0.68 Mb	16
	Mean distance to social zone	chr4:150582701	5.884	1.1 Mb	19
	Total distance to object zone	chr19:20667417	6.619	1.6 Mb	23
	Total distance to social zone	chr19:55339863	6.643	0.68 Mb	16
	Total distance to social zone	chr4:150582701	5.788	1.1 Mb	19
	Total travel distance	chr14:34908176	5.648	0.74 Mb	10
	Total travel distance	chr14:41727329	5.627	0.85 Mb	5

**Figure 3:**
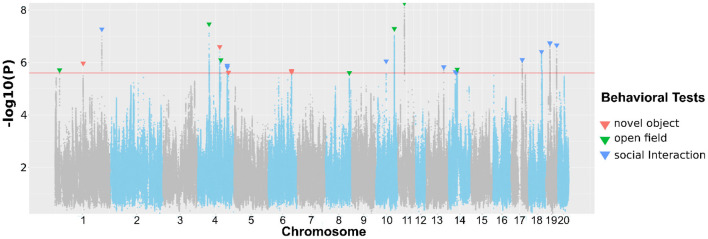
Association of approximately 3 million SNPs with behavioral traits measured in OFT, NOIT, or SIT. The red horizontal line denotes the *p*-value for reaching genome-wide significance. The downward arrows denote the SNPs with the largest –log10(P) for each genome-wide significant association.

**Figure 4:**
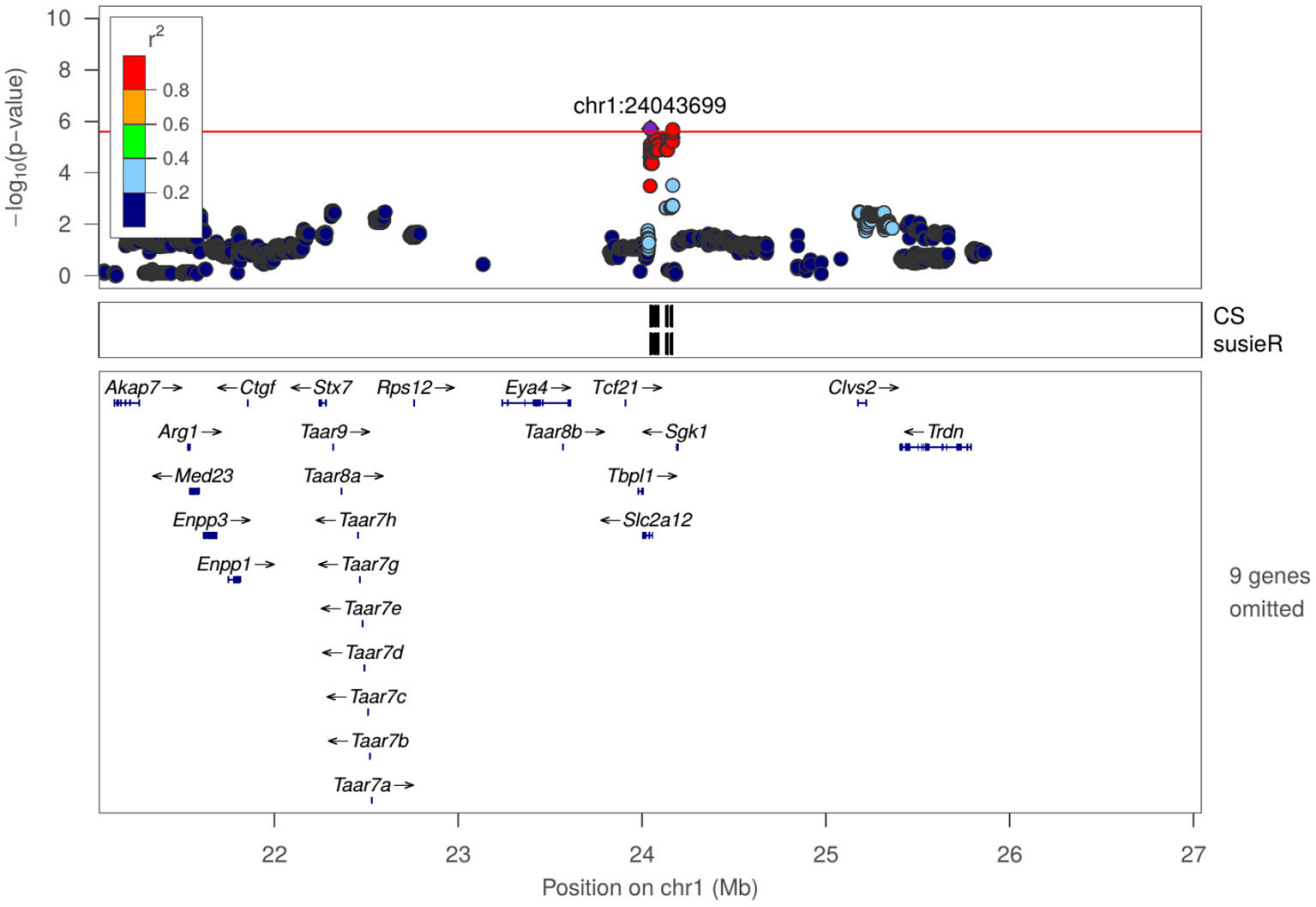
Regional association plot for frequency of entering center zone in OFT at chr1:24043699.

**Figure 5:**
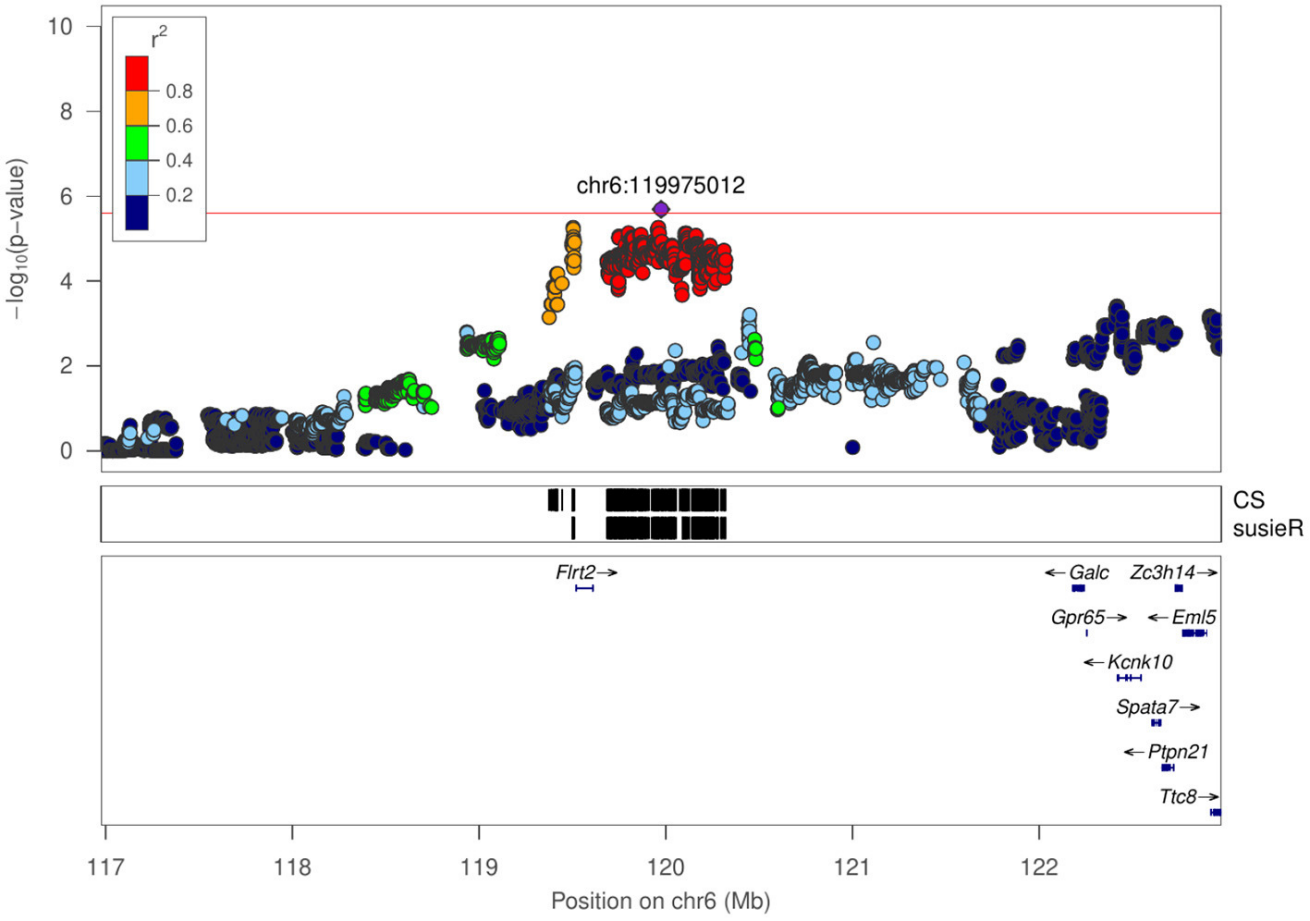
Regional association plot for mean distance to center zone in NOIT at chr6:119975012.

**Figure 6:**
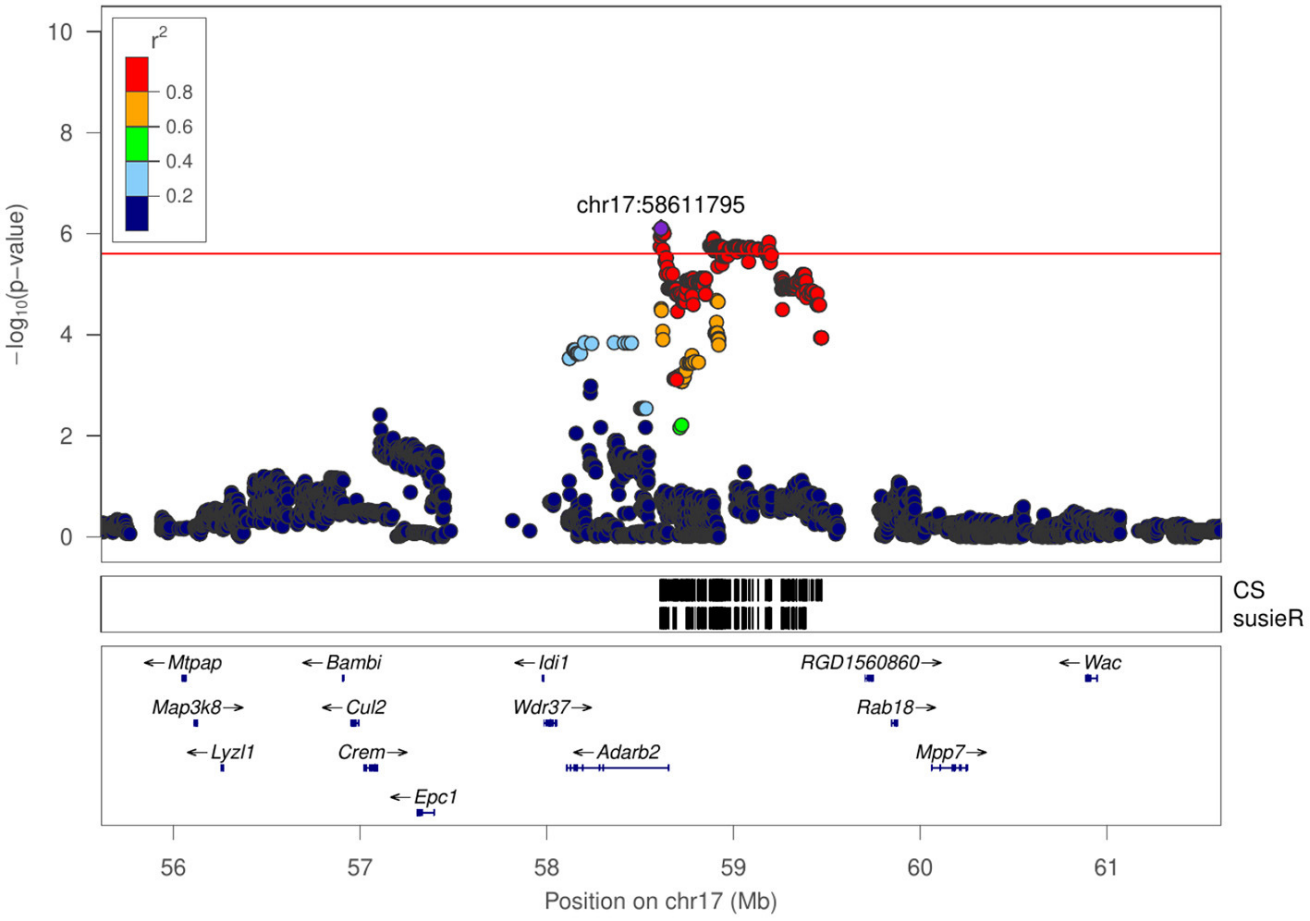
Regional association plot for latency of entering social zone in SIT at chr17:58611795.

### 3.5. Pleiotropic Loci

To determine if traits that mapped to the same location are pleiotropic, we considered the minor allele frequency (MAF), and the strain distribution pattern (SDP) of the most significant SNP among the 8 founder strains that were used to create the HS. Using these criteria, we did not observe any pleiotropic loci between the traits analyzed in different tests. However, we did identify pleiotropic loci between the traits of the same behavior test. Most of these traits are highly correlated, as shown in Figure 1. With the exception of three sets of QTL (Supplementary Table S3), all others share the same top SNP (Supplementary Table S2).

### 3.6. Candidate Gene Identification

The number of genes within the identified QTL ranges from 1 to 127 (mean: 30.1, median: 19, Table 2). There is only one region that contains a single gene: *Adarb2* within chr17:58Mb for latency of entering social zone in SIT. However, it is also possible that the causal allele is a regulatory variant that is located in this interval but regulates a gene outside of the identified interval.

All other loci contained more than one gene. To identify candidate genes, we combined several criteria: (1) located in the credible set identified by either one of the fine mapping methods. (2) the presence of moderate or high impact variants located within the gene, as predicted by SnpEff (40). We also require these variants are in high LD with the top SNP. We identified 149 coding variants within 30 QTL, 8 of which were predicted to have a high impact (Supplementary Table S4). (3) the presence of a significant cis-eQTL in one or more of the five brain regions in a dataset containing 88 navie adult HS rats (39), (4) has a human ortholog that has been reported to be associated with psychiatric diseases (including drug abuse). When multiple candidates are present using the above criteria, we remove the gene with very low expression levels across all five regions in the RNA-seq data set (e.g., FPKM < 0.5) and select the candidate with the strongest support for the literature. Combining these criteria with a literature search conducted using GeneCup (41), we identified plausible candidate genes within 17 loci (Table 3).

**Table 3:** Candidate genes.

**Test**	**Trait**	**Top SNP**	**Candidate gene**	**Supporting evidence**	**Human GWAS**	**Expression level (FPKM)**	**Gene function**
OFT	Frequency of entering center zone, Total distance to center zone	chr4:118013062	Cyp26b1	Missense variants, cis-eQTL in IL and PL	Schizophrenia (42, 43)	IL 7.29 ± 2.26	Inactivate all-trans retinoic acid (44)
OFT	Total distance to center zone	chr14:44904830	Fam114a1	Missense variants, cis-eQTL in LHb	Alcohol consumption measurement (45)	LHb 3.72 ± 0.77	Also known as Noxp20, neuronal cell development (46)
OFT	Total travel distance	chr10:94549701	Cacng4	cis-eQTL in Acbc	Bipolar disorder and Schizophrenia (47)	Acbc 55.22 ± 4.79	Calcium channel (48)
OFT	Total distance to center zone	chr4:58009499	Ube2h	cis-eQTL in OFC	Unipolar depression, mood disorder (49)	OFC 49.22 ± 2.72	Ubiquination of proteins (50)
NOIT	Duration in center zone, distance to center zone	chr4:112234344	Eva1a	Missense variants, cis-eQTL in PL, IL and OFC		LHb 6.74 ± 1.69	Formation of the autophagosome (51)
NOIT	Total distance to center zone	chr1:144080083	Sec11a	cis-eQTL in PL and IL	Unipolar depression, depressive symptom measurement, response to ketamine, bipolar disorder, schizophrenia (43, 52, 53)	Acbc 33.34 ± 3.73	Metabolism of proteins (54)
NOIT	Total distance to center zone	chr4:156801420	Mlf2	cis-eQTL in PL	Smoking status measurement (55)	Acbc 307.85 ± 40.01	Molecular chaperone in multi-protein complex assembly, signaling transduction, and endocytosis (56).
SIT	Duration in object zone	chr18:65869186	Rab27b	missense variants, cis-eQTL in LHb	Unipolar depression, bipolar disorder (57–59)	Acbc 8.03 ± 2.7	Vesicular fusion and trafficking (60)
SIT	Duration in social zone, distance to social zone	chr4:151128675	Ankrd26	Missense variants	smoking initiation (61)	IL 4.75 ± 1.21	Cell signaling (62)
SIT	Frequency of entering object zone	chr13:90335374	Kcnj9	Missense variants, cis-eQTL in IL, PL and OFC	Alcohol consumption measurement (63)	OFC 65.46 ± 6.64	Adult neurogenesis (64), cocaine addiction (65)
SIT	Frequency of entering object zone	chr1:239076581	Gda	cis-eQTL in IL	General cognitive function (66)	Acbc 84.67 ± 16.92	Cypin, cytoplasmic PSD95 Interactor (67)
SIT	Latency of entering social zone	chr10:52831274	Dnah9	Missense variants	Schizophrenia (68)	LH 3.59 ± 1.33	Component of microtubule (69)
SIT	Latency of entering social zone	chr17:58611795	Adarb2	cis-eQTL in Acbc and LH	Unipolar depression, smoking status measurement, systolic blood pressure (58, 70)	PL 2.22 ± 0.68	Editing of neurotrasmiter mRNA (71)
SIT	Distance to social zone	chr19:55339863	Ctu2	Missense variants, cis-eQTL in IL	Autism spectrum disorder symptom (72)	LHb 6.39 ± 1.14	Post-transcriptional modification of tRNAs (73)
SIT	Distance to social zone	chr4:150582701	Cacna1c		Schizophrenia (74), biopolor disorder (75)	PL 6.76 ± 1.49	Calcium channel (76)
SIT	Total travel distance	chr14:34908176	Clock	Missense variants, cis-eQTL in LHb		Acbc 11.15 ± 1.82	Regulate circadian rhythms (77, 78)
SIT	Total travel distance	chr14:41727329	Grxcr1	cis-eQTL in Acbc and LHb	Cognitive decline in depression (79)	IL 2.97 ± 1.02	S-glutathionylation of proteins (80)

*Acbc, Accubens core; IL, infralimbic cortex; LHb, lateral habenular; OFC, orbitofrontal; PL, prelimbic cortex*.

In addition, for total distance to the novel object zone, the QTL region on chr1 (144 Mb, size: 4.1 Mb, Supplementary Figure S4) contains 69 gene with human orthologs. We found 14 of these genes have been reported in human GWAS to be associated with psychiatric conditions or addiction with genome-wide significance (*ACAN, ADAMTSL3, ALPK3, CPEB1, FES, FURIN, LINC00933, MIR9-3HG, MRPL46, NMB, POLG-DT, SEC11A, ZNF592, and ZSCAN2*, Supplementary Table S5). Three additional genes with sub-threshold significance in human GWAS are also included. These genes are all located in a syntenic region on human chromosome 15 (82.5–90.8 Mb). Although based on the criteria described above, *Sec11a* is the best candidate gene (Table 3), it is possible that this region contains multiple genes that are associated with the trait.

## 4. Discussion

As part of a GWAS on intravenous nicotine self-administration in adolescent HS rats that we are conducting (28, 81), we collected several behavioral phenotypes related to anxiety, novelty exploration, and social interaction. We have previously reported that these behavioral traits contribute to the variation in nicotine intake (28). We report here GWAS results of three behavioral traits: OFT, NOIT, and SIT, which were all conducted in the same open field. We identified 30 QTLs for 23 traits. Using a set criteria outlined above, we identified 17 candidate genes.

OFT, NOIT, and SIT are widely used behavioral assays in rodents. With over 1,200 rats, ours represent some of the largest data collected using these assays. Similar to our interim report on this data set (28), we found a large number of correlations with relatively low coefficients (e.g., *r* < 0.4) but with high statistical significance. It is likely that these correlated traits are controlled by the same behavioral processes and thus are influenced by the same genetic factors. In fact, our genetic analysis did find several pleiotropic sites (Supplementary Table S3). Almost all pleiotropic loci are reported for traits measured in the same behavior assay. It is likely that further increasing sample size will provide greater statistical power to detect pleiotropic effect across different behavioral assays.

Many of the candidate genes in this study have been associated with psychiatric or drug abuse traits in humans. For example, we identified *Cyp26b1*, a retinoic acid degrading enzyme, as a candidate gene for the frequency of entering the center zone and total distance to the center zone in OFT; both of which are measures of anxiety-like behaviors (rats with more anxiety-like behavior would enter the center zone less frequntly and have smaller distance to the center zone) (11). *Cyp26b1* has been associated with Schizophrenia in several human GWAS (42, 43). Anxiety symptoms are common in schizophrenia patients (82, 83). *Cyp26b1* is expressed in parvalbumin-positive interneurons (84). Most interestingly, knockdown *Cyp26b1* in the nucleus accumbens shell decreased anxiety-like behavior (44).

Among the candidate genes for NOIT, *Eva1a* is a candidate gene for the duration stayed in the center zone that contained the novel object. *Eva1a* is supported by strong cis-eQTL and a missense variant but has no literature support. Thus, further evaluating the role of *Evala* could potentially lead to new mechanisms for novelty seeking-like behavior. *Sec11a*, a candidate gene for total distance in the center zone, is associated with depression and schizophrenia (43, 52, 53). *Mlf2*, a candidate gene for total distance to center zone in NOIT, is associated with smoking in humans (55) and has very high expression levels in the accumbens (Table 3).

For the SIT, we identified *Cacna1c*, encoding the Ca_*v*_1.2 subunit of the L-type Ca^2+^ channel, as a candidate gene for distance to the social zone, where the stimulus rat resided. *Cacna1c* has been associated with schizophrenia (74) and bipolar disorder (75) in human GWAS. Both schizophrenia and bipolar disorders are associated with impairments in a range of social deficits (85, 86). In animal studies, Sprague-Dawley rats with heterozygotic deletion of the *Cacna1c* gene (homozygotic mutation is lethal) showed many deficits in social behavior. These included reduced levels of ultrasonic vocalizations during rough-and-tumble play, as well as social approach behavior elicited by playback of ultrasonic vocalizations (87, 88). In mice, a knockdown of *Cacna1c* in the nucleus accumbens significantly increased susceptibility to social stress (89). Knocking down of *Cacna1c* in the prefrontal cortex of adult mice also recapitulated many of the social deficits (90). Importantly, some of the behavioral effects of *Cacna1c* appear to interact with genetic background (91).

Among the other candidate genes for the SIT traits, *Rab27b* is involved in the presynaptic mechanism of long-term potentiation (92) as well as myelin biogenesis in oligodendrocytes (93). *Ankrd26* is expressed in the arcuate and ventromedial nuclei and in the ependyma*Gda*, also known as Cypin, in located in the postsynaptic density (95). *Ctu2* is involved in post-translational modification of tRNAs (73). *Adarb2* has been associated with home cage activity (96) and unipolar depression (58). The *Clock* gene is involved in the maintenance of locomotor rhythms (97). Mutations of the *CLOCK* gene have been implicated in many psychiatric disorders (98). Although these candidates are well supported by multiple lines of evidence, additional work is needed to confirm their causal relationship to the corresponding behavioral traits.

The total distance to the novel object zone is associated with chr1:144080083 (allele frequency: 0.91, −*log*_10_(*p*) = 5.969, size of interval: 4.1 Mb, Supplementary Figure S34). This SNP is also associated with the duration rats stayed in the novel object zone, although the *p*-value did not reach genome-wide significance (-logP = 4.63). This region contains 69 known genes. Its syntenic region on human Chr15 (82.5-90.8 Mb) is a hotspot for human pyschiatric diseases, containing 30 SNPs and 14 genes (*ACAN, ADAMTSL3, ALPK3, CPEB1, FES, FURIN, LINC00933, MIR9-3HG, MRPL46, NMB, POLG-DT, SEC11A, ZNF592, and ZSCAN2*) associated with generalized anxiety disorder, schizophrenia, bipolar disorder, obsessive compulsive disorder, attentions deficit hyperactivity disorder, autism spectrum disorder, and unipolar depression, smoking behavior, etc. These results are reported in 21 publications (Supplementary Table S5). Using the criteria described above, we identified *Sec11a* as the best candidate gene (Table 3). However, given the large number of genetic variants reported in human GWAS that are associated with psychiatric conditions within this syntenic region, it is very likely that this region contains multiple genes that are associated with novelty seeking-like behavior.

We include overlapping with human psychiatric GWAS results as part of the criteria in prioritizing candidate genes. It is possible that this approach could introduce bias and prevent us from making novel discoveries. For example, two (*Cyp26b1* and *Cacng4*) of the four candidate genes for OFT have been associated with schizophrenia, rather than anxiety. However, many genetic variants are pleiotropic for multiple psychiatric diseases (99). For example, polygenic risk scores for schizophrenia have been associated with many other psychiatric diseases, such as anxiety disorder (100) or major depressive disorder (101), or cognitive performance (102). Together with other evidence, we believe considering human psychiatric GWAS findings when identifying candidate genes in our study, even when the behavior trait in rats does not map directly to the psychiatric disease, is still valid and will likely increase the translational value of our findings.

The presence of cis-eQTL in the brain is one of the strongest pieces of evidence that we use to prioritizes candidate genes. Fourteen of the 17 candidate genes we identified have cis-eQTL. Seven of these genes also contained missense mutations, which further support their biological function related to the phenotype they are associated with. The only candidate gene that is not support by either cis-eQTL or missense mutation is *Cacna1c*, associated with distance to the social zone. However, the role of *Cacna1c* in social behavior has been well documented in rats and mice (87–90). We also required all candidate genes located in regions confirmed by fine mapping. For example, the *Crhr1* gene, which encodes corticotrophin release hormone receptor 1, is located in the locus for total travel distance in the OFT but is not supported by fine mapping. Dispite strong literature support for the role of *Crhr1* in anxiety-like behavior in OFT in rats (103, 104) and mice (105), we nominated a different gene, *Cacng4*, to be the candidate gene for this locus. We anticipate further improvement in the statistical power of eQTL data and the availability of additional functional genomics data, such as 3D chromatin interaction (106, 107), will help us to identify additional candidate genes.

The HS rat population has already been successfully used in genetic mapping studies of physiological or behavioral traits (24, 25, 27). Prior study mapped several anxiety-like traits using zero maze (23). GWAS using HS to study behavioral regulation (108), response to cocaine cues (109), cocaine (110), nicotine (28, 81), or oxycodone (111) self-administration are underway. Our study adds to the literature 30 QTLs and 17 candidate genes for psychiatric related behavioral traits. Although we prioritized candidate gene selection based on functional genomics evidence, most of the candidate genes we identified have strong literature support for their role in human psychiatric diseases. This suggests that the rest of the candidate genes likely represent novel findings that can be the catalyst for future molecular and genetic insights on psychiatric diseases. In addition, these findings provide strong support for the use of the HS population in study psychiatric disorders.

## Data Availability Statement

The original contributions presented in the study are publicly available. This data can be found through the C-GORD database at doi: 10.48810/P44W2 and through https://www.genenetwork.org.

## Ethics Statement

The animal study was reviewed and approved by University Tennessee Health Science Center IACUC.

## Author Contributions

HC and AP designed the study. TW and AG collected the data. AC, OP, and MG analyzed the data. MG, AP, and HC wrote the manuscript. All authors contributed to the article and approved the submitted version.

## Funding

This work was supported by the National Institute on Drug Abuse (P50 DA037844).

## Conflict of Interest

The authors declare that the research was conducted in the absence of any commercial or financial relationships that could be construed as a potential conflict of interest.

## Publisher's Note

All claims expressed in this article are solely those of the authors and do not necessarily represent those of their affiliated organizations, or those of the publisher, the editors and the reviewers. Any product that may be evaluated in this article, or claim that may be made by its manufacturer, is not guaranteed or endorsed by the publisher.

## References

[1] Hamidullah S Thorpe H Frie J Mccurdy R Khokhar J. Adolescent substance use and the brain: behavioral, cognitive and neuroimaging correlates. Front Hum Neurosci. (2020) 14:298. 10.3389/fnhum.2020.00298 32848673 PMC7418456

[2] Garey L Olofsson H Garza T Shepherd J Smit T Zvolensky M. The role of anxiety in smoking onset, severity, and cessation-related outcomes: a review of recent literature. Curr Psychiatry Rep. (2020) 22:38. 10.1007/s11920-020-01160-5 32506166

[3] DuPont R. Anxiety and addiction: a clinical perspective on comorbidity. Bull Menninger Clin. (1995) 59(2 Suppl A):53–72. 7795572

[4] Laucht M Becker K El-Faddagh M Hohm E Schmidt M. Association of the DRD4 exon III polymorphism with smoking in fifteen-year-olds: a mediating role for novelty seeking? J Am Acad Child Adolesc Psychiatry. (2005) 44:477–84. 10.1097/01.chi.0000155980.01792.7f 15843770

[5] Mahoney J Thompson-Lake D Cooper K Verrico C Newton T De La Garza R. A comparison of impulsivity, depressive symptoms, lifetime stress and sensation seeking in healthy controls versus participants with cocaine or methamphetamine use disorders. J Psychopharmacol. (2015) 29:50–6. 10.1177/0269881114560182 25424624

[6] Truan F. Addiction as a social construction: a postempirical view. J Psychol. (1993) 127:489–99. 10.1080/00223980.1993.9914886 8271227

[7] Wilking J Hesterberg K Nguyen V Cyboron A Hua A Stitzel J. Comparison of nicotine oral consumption and baseline anxiety measures in adolescent and adult C57BL/6J and C3H/Ibg mice. Behav Brain Res. (2012) 233:280–7. 10.1016/j.bbr.2012.05.022 22633961 PMC3402682

[8] Manhães A Guthierrez M Filgueiras C Abreu-Villaça Y. Anxiety-like behavior during nicotine withdrawal predict subsequent nicotine consumption in adolescent C57BL/6 mice. Behav Brain Res. (2008) 193:216–24. 10.1016/j.bbr.2008.05.018 18573281

[9] Redolat R Pérez-Martínez A Carrasco M Mesa P. Individual differences in novelty-seeking and behavioral responses to nicotine: a review of animal studies. Curr Drug Abuse Rev. (2009) 2:230–42. 10.2174/1874473710902030230 20443770

[10] Pelloux Y Giorla E Montanari C Baunez C. Social modulation of drug use and drug addiction. Neuropharmacology. (2019) 159:107545. 10.1016/j.neuropharm.2019.02.027 30807753

[11] Prut L Belzung C. The open field as a paradigm to measure the effects of drugs on anxiety-like behaviors: a review. Eur J Pharmacol. (2003) 463:3–33. 10.1016/S0014-2999(03)01272-X 12600700

[12] Kraeuter AK Guest PC Sarnyai Z. The open field test for measuring locomotor activity and Anxiety-Like behavior. Methods Mol. Biol. (2019) 1916:99–103. 10.1007/978-1-4939-8994-2_9 30535687

[13] Kulesskaya N Voikar V. Assessment of mouse anxiety-like behavior in the light-dark box and open-field arena: role of equipment and procedure. Physiol Behav. (2014) 133:30–8. 10.1016/j.physbeh.2014.05.006 24832050

[14] Seibenhener ML Wooten MC. Use of the open field maze to measure locomotor and anxiety-like behavior in mice. J Vis Exp. (2015) 96:e52434. 10.3791/52434 25742564 PMC4354627

[15] Flagel SB Waselus M Clinton SM Watson SJ Akil H. Antecedents and consequences of drug abuse in rats selectively bred for high and low response to novelty. Neuropharmacology. (2014) 76(Pt B):425–36. 10.1016/j.neuropharm.2013.04.033 23639434 PMC3766490

[16] Bardo MT Donohew RL Harrington NG. Psychobiology of novelty seeking and drug seeking behavior. Behav Brain Res. (1996) 77:23–43. 10.1016/0166-4328(95)00203-0 8762157

[17] Belin D Berson N Balado E Piazza PV Deroche-Gamonet V. High-novelty-preference rats are predisposed to compulsive cocaine self-administration. Neuropsychopharmacology. (2011) 36:569–79. 10.1038/npp.2010.188 20980989 PMC3055686

[18] Cain ME Saucier DA Bardo MT. Novelty seeking and drug use: contribution of an animal model. Exp Clin Psychopharmacol. (2005) 13:367–75. 10.1037/1064-1297.13.4.367 16366767

[19] Toth I Neumann ID. Animal models of social avoidance and social fear. Cell Tissue Res. (2013) 354: 107–118. 10.1007/s00441-013-1636-4 23760888

[20] File SE Seth P. A review of 25 years of the social interaction test. Eur J Pharmacol. (2003) 463:35–53. 10.1016/S0014-2999(03)01273-1 12600701

[21] File S Cheeta S Irvine E Tucci S Akthar M. Conditioned anxiety to nicotine. Psychopharmacology (Berl). (2002) 164:309–17. 10.1007/s00213-002-1219-7 12424555

[22] Hansen C Spuhler K. Development of the national institutes of health genetically heterogeneous rat stock. Alcohol Clin Exp Res. (1984) 8:477–9. 10.1111/j.1530-0277.1984.tb05706.x 6391259

[23] Baud A Hermsen R Guryev V Stridh P Graham D McBride M. Combined sequence-based and genetic mapping analysis of complex traits in outbred rats. Nat Genet. (2013) 45:767–75. 10.1038/ng.2644 23708188 PMC3821058

[24] Woods L Mott R. Heterogeneous stock populations for analysis of complex traits. Methods Mol Biol. (2017) 1488:31–44. 10.1007/978-1-4939-6427-7_2 27933519 PMC5869698

[25] Solberg Woods L Palmer A. Using heterogeneous stocks for fine-mapping genetically complex traits. Methods Mol Biol. (2019) 2018:233–47. 10.1007/978-1-4939-9581-3_11 31228160 PMC9121584

[26] Chitre AS Polesskaya O Holl K Gao J Cheng R Bimschleger H. Genome-Wide association study in 3,173 outbred rats identifies multiple loci for body weight, adiposity, and fasting glucose. Obesity. (2020) 28:1964–73. 10.1002/oby.22927 32860487 PMC7511439

[27] Keele G Prokop J He H Holl K Littrell J Deal A. Genetic fine-mapping and identification of candidate genes and variants for adiposity traits in outbred rats. Obesity (Silver Spring). (2018) 26:213–22. 10.1002/oby.22075 29193816 PMC5740008

[28] Wang T Han W Chitre A Polesskaya O Solberg Woods L Palmer A. Social and anxiety-like behaviors contribute to nicotine self-administration in adolescent outbred rats. Sci Rep. (2018) 8:18069. 10.1038/s41598-018-36263-w 30584246 PMC6305389

[29] Kessler R Amminger G Aguilar-Gaxiola S Alonso J Lee S Ustün T. Age of onset of mental disorders: a review of recent literature. Curr Opin Psychiatry. (2007) 20:359–64. 10.1097/YCO.0b013e32816ebc8c 17551351 PMC1925038

[30] Gileta A Gao J Chitre A Bimschleger H St Pierre C Gopalakrishnan S. Adapting genotyping-by-sequencing and variant calling for heterogeneous stock rats. G3 (Bethesda). (2020) 10:2195–205. 10.1534/g3.120.401325 32398234 PMC7341140

[31] Yang J Benyamin B McEvoy B Gordon S Henders A Nyholt D. Common SNPs explain a large proportion of the heritability for human height. Nat Genet. (2010) 42:565–9. 10.1038/ng.608 20562875 PMC3232052

[32] Yang J Lee S Goddard M Visscher P. GCTA: a tool for genome-wide complex trait analysis. Am J Hum Genet. (2011) 88:76–82. 10.1016/j.ajhg.2010.11.011 21167468 PMC3014363

[33] Cheng R Parker C Abney M Palmer A. Practical considerations regarding the use of genotype and pedigree data to model relatedness in the context of genome-wide association studies. G3 (Bethesda). (2013) 3:1861–7. 10.1534/g3.113.007948 23979941 PMC3789811

[34] Gonzales N Seo J Hernandez Cordero A St Pierre C Gregory J Distler M. Genome wide association analysis in a mouse advanced intercross line. Nat Commun. (2018) 9:5162. 10.1038/s41467-018-07642-8 30514929 PMC6279738

[35] Cheng R Palmer A. A simulation study of permutation, bootstrap, and gene dropping for assessing statistical significance in the case of unequal relatedness. Genetics. (2013) 193:1015–8. 10.1534/genetics.112.146332 23267053 PMC3583989

[36] Maller J McVean G Byrnes J Vukcevic D Palin K Su Z. Bayesian refinement of association signals for 14 loci in 3 common diseases. Nat Genet. (2012) 44:1294–301. 10.1038/ng.2435 23104008 PMC3791416

[37] Wang G Sarkar A Carbonetto P Stephens M. A simple new approach to variable selection in regression, with application to genetic fine mapping. J R Stat Soc B. (2020) 82:1273–300. 10.1111/rssb.12388 PMC10201948 37220626

[38] Hukku A Pividori M Luca F Pique-Regi R Im H Wen X. Probabilistic colocalization of genetic variants from complex and molecular traits: promise and limitations. Am J Hum Genet. (2021) 108:25–35. 10.1016/j.ajhg.2020.11.012 33308443 PMC7820626

[39] Munro D Wang T Chitre AS Polesskaya O Saba L Chen H. Mapping genotype-expression associations in Heterogeneous Stock rat brains to advance behavioral genetics research. In: Genetics and Epigenetics Cross-Cutting Research Team (GECCRT) Meeting. (Washington, D.C.), (2021).

[40] Cingolani P Platts A Wang lL Coon M Nguyen T Wang L. A program for annotating and predicting the effects of single nucleotide polymorphisms, SnpEff: SNPs in the genome of Drosophila melanogaster strain w1118; iso-2; iso-3. Fly (Austin). (2012) 6:80–92. 10.4161/fly.19695 22728672 PMC3679285

[41] Gunturkun MH Flashner E Wang T Mulligan MK Williams RW Prins P. GeneCup: mine PubMed for gene relationships using custom ontology and deep learning. bioRxiv. (2021) 10.1101/2020.09.17.297358

[42] Schizophrenia Working Group of the Psychiatric Genomics Consortium. Biological insights from 108 schizophrenia-associated genetic loci. Nature. (2014) 511:421–7. 10.1038/nature13595 25056061 PMC4112379

[43] Goes F McGrath J Avramopoulos D Wolyniec P Pirooznia M Ruczinski I. Genome-wide association study of schizophrenia in ashkenazi jews. Am J Med Genet B Neuropsychiatr Genet. (2015) 168:649–59. 10.1002/ajmg.b.32349 26198764

[44] Zhang Y Crofton E Smith T Koshy S Li D Green T. Manipulation of retinoic acid signaling in the nucleus accumbens shell alters rat emotional behavior. Behav Brain Res. (2019) 376:112177. 10.1016/j.bbr.2019.112177 31449909 PMC7359447

[45] Gelernter J Kranzler H Sherva R Almasy L Koesterer R Smith A. Genome-wide association study of alcohol dependence:significant findings in African- and European-Americans including novel risk loci. Mol Psychiatry. (2014) 19:41–9. 10.1038/mp.2013.145 24166409 PMC4165335

[46] Boucquey M De Plaen E Locker M Poliard A Mouillet-Richard S Boon T. Noxp20 and Noxp70, two new markers of early neuronal differentiation, detected in teratocarcinoma-derived neuroectodermic precursor cells. J Neurochem. (2006) 99:657–69. 10.1111/j.1471-4159.2006.04093.x 17029606

[47] Curtis D Vine A McQuillin A Bass N Pereira A Kandaswamy R. Case-case genome-wide association analysis shows markers differentially associated with schizophrenia and bipolar disorder and implicates calcium channel genes. Psychiatr Genet. (2011) 21:1–4. 10.1097/YPG.0b013e3283413382 21057379 PMC3024533

[48] Kious B Baker C Bronner-Fraser M Knecht A. Identification and characterization of a calcium channel gamma subunit expressed in differentiating neurons and myoblasts. Dev Biol. (2002) 243:249–59. 10.1006/dbio.2001.0570 11884034

[49] Li Q Tian C Seabrook G Drevets W Narayan V. Analysis of 23andMe antidepressant efficacy survey data: implication of circadian rhythm and neuroplasticity in bupropion response. Transl Psychiatry. (2016) 6:e889. 10.1038/tp.2016.171 27622933 PMC5048209

[50] Vourc'h P Martin I Bonnet-Brilhault F Marouillat S Barthélémy C Pierre Müh J. Mutation screening and association study of the UBE2H gene on chromosome 7q32 in autistic disorder. Psychiatr Genet. (2003) 13:221–5. 10.1097/00041444-200312000-00005 14639049

[51] Hu J Li G Qu L Li N Liu W Xia D. TMEM166/EVA1A interacts with ATG16L1 and induces autophagosome formation and cell death. Cell Death Dis. (2016) 7:e2323. 10.1038/cddis.2016.230 27490928 PMC5108317

[52] Guo W Machado-Vieira R Mathew S Murrough J Charney D Grunebaum M. Exploratory genome-wide association analysis of response to ketamine and a polygenic analysis of response to scopolamine in depression. Transl Psychiatry. (2018) 8:280. 10.1038/s41398-018-0311-7 30552317 PMC6294748

[53] Pardiñas A Holmans P Pocklington A Escott-Price V Ripke S Carrera N. Common schizophrenia alleles are enriched in mutation-intolerant genes and in regions under strong background selection. Nat Genet. (2018) 50:381–9. 10.1038/s41588-018-0059-2 29483656 PMC5918692

[54] Fine A Irihimovitch V Dahan I Konrad Z Eichler J. Cloning, expression, and purification of functional Sec11a and Sec11b, type I signal peptidases of the archaeon Haloferax volcanii. J Bacteriol. (2006) 188:1911–9. 10.1128/JB.188.5.1911-1919.2006 16484202 PMC1426568

[55] Liu M Jiang Y Wedow R Li Y Brazel D Chen F. Association studies of up to 1.2 million individuals yield new insights into the genetic etiology of tobacco and alcohol use. Nat Genet. (2019) 51:237–44. 10.1038/s41588-018-0307-5 30643251 PMC6358542

[56] Yang J Cao D Zhang Y Ou R Yin Z Liu Y. The role of phosphorylation of MLF2 at serine 24 in BCR-ABL leukemogenesis. Cancer Gene Ther. (2020) 27:98–107. 10.1038/s41417-019-0152-4 31831854

[57] Coleman J Gaspar H Bryois J Breen G. The genetics of the mood disorder spectrum: genome-wide association analyses of more than 185,000 cases and 439,000 controls. Biol Psychiatry. (2020) 88:169–84. 10.1016/j.biopsych.2019.10.015 31926635 PMC8136147

[58] Howard D Adams M Clarke T Hafferty J Gibson J Shirali M. Genome-wide meta-analysis of depression identifies 102 independent variants and highlights the importance of the prefrontal brain regions. Nat Neurosci. (2019) 22:343–52. 10.1038/s41593-018-0326-7 30718901 PMC6522363

[59] Wray N Ripke S Mattheisen M Trzaskowski M Byrne E Abdellaoui A. Genome-wide association analyses identify 44 risk variants and refine the genetic architecture of major depression. Nat Genet. (2018) 50:668–81. 10.1038/s41588-018-0090-3 29700475 PMC5934326

[60] Chen D Guo J Miki T Tachibana M Gahl W. Molecular cloning and characterization of rab27a and rab27b, novel human rab proteins shared by melanocytes and platelets. Biochem Mol Med. (1997) 60:27–37. 10.1006/bmme.1996.2559 9066979

[61] Brazel D Jiang Y Hughey J Turcot V Zhan X Gong J. Exome chip meta-analysis fine maps causal variants and elucidates the genetic architecture of rare coding variants in smoking and alcohol use. Biol Psychiatry. (2019) 85:946–55. 10.1016/j.biopsych.2018.11.024 30679032 PMC6534468

[62] Bera T Liu X Yamada M Gavrilova O Mezey E Tessarollo L. A model for obesity and gigantism due to disruption of the Ankrd26 gene. Proc Natl Acad Sci USA. (2008) 105:270–5. 10.1073/pnas.0710978105 18162531 PMC2224199

[63] Sanchez-Roige S Fontanillas P Elson S Gray J de Wit H Davis L. Genome-wide association study of alcohol use disorder identification test (AUDIT) scores in 20 328 research participants of European ancestry. Addict Biol. (2019) 24:121–31. 10.1111/adb.12574 29058377 PMC6988186

[64] Kempermann G Chesler E Lu L Williams R Gage F. Natural variation and genetic covariance in adult hippocampal neurogenesis. Proc Natl Acad Sci USA. (2006) 103:780–5. 10.1073/pnas.0510291103 16407118 PMC1325968

[65] Huggett S Stallings M. Genetic architecture and molecular neuropathology of human cocaine addiction. J Neurosci. (2020) 40:5300–13. 10.1523/JNEUROSCI.2879-19.2020 32457073 PMC7329314

[66] Davies G Lam M Harris S Trampush J Luciano M Hill W. Study of 300,486 individuals identifies 148 independent genetic loci influencing general cognitive function. Nat Commun. (2018) 9:2098. 10.1038/s41467-018-04362-x 29844566 PMC5974083

[67] Patel M Swiatkowski P Kwon M Rodriguez A Campagno K Firestein B. A novel short isoform of cytosolic PSD-95 Interactor (Cypin) regulates neuronal development. Mol Neurobiol. (2018) 55:6269–81. 10.1007/s12035-017-0849-z 29294243 PMC6028325

[68] Liu L Gu H Hou F Xie X Li X Zhu B. Dyslexia associated functional variants in Europeans are not associated with dyslexia in Chinese. Am J Med Genet B Neuropsychiatr Genet. (2019) 180:488–95. 10.1002/ajmg.b.32750 31264768

[69] Olczak M Poniatowski L Kwiatkowska M Samojlowicz D Tarka S Wierzba-Bobrowicz T. Immunolocalization of dynein, dynactin, and kinesin in the cerebral tissue as a possible supplemental diagnostic tool for traumatic brain injury in postmortem examination. Folia Neuropathol. (2019) 57:51–62. 10.5114/fn.2019.83831 31038188

[70] Sung Y Winkler T de Las Fuentes L Bentley A Brown M Kraja A. A large-scale multi-ancestry genome-wide study accounting for smoking behavior identifies multiple significant loci for blood pressure. Am J Hum Genet. (2018) 102:375–400. 10.1016/j.ajhg.2018.01.015 29455858 PMC5985266

[71] Mittaz L Antonarakis S Higuchi M Scott H. Localization of a novel human RNA-editing deaminase (hRED2 or ADARB2) to chromosome 10p15. Hum Genet. (1997) 100:398–400. 10.1007/s004390050523 9272162

[72] Chaste P Klei L Sanders S Hus V Murtha M Lowe J. A genome-wide association study of autism using the simons simplex collection: does reducing phenotypic heterogeneity in autism increase genetic homogeneity? Biol Psychiatry. (2015) 77:775–84. 10.1016/j.biopsych.2014.09.017 25534755 PMC4379124

[73] Erratum. Hum Mutat. (2021) 42:219. 10.1002/humu.24157 33559988

[74] Liu Y Wu X Xia X Yao J Wang B. The genome-wide supported CACNA1C gene polymorphisms and the risk of schizophrenia: an updated meta-analysis. BMC Med Genet. (2020) 21:159. 10.1186/s12881-020-01084-0 32770953 PMC7414708

[75] Mosheva M Serretti A Stukalin Y Fabbri C Hagin M Horev S. Association between CANCA1C gene rs1034936 polymorphism and alcohol dependence in bipolar disorder. J Affect Disord. (2020) 261:181–6. 10.1016/j.jad.2019.10.015 31634677

[76] Yamakage M Namiki A. Calcium channels-basic aspects of their structure, function and gene encoding; anesthetic action on the channels-a review. Can J Anaesth. (2002) 49:151–64. 10.1007/BF03020488 11823393

[77] Mendoza J. Food intake and addictive-like eating behaviors: time to think about the circadian clock(s). Neurosci Biobehav Rev. (2019) 106:122–32. 10.1016/j.neubiorev.2018.07.003 29990504

[78] Salaberry N Mendoza J. Insights into the role of the habenular circadian clock in addiction. Front Psychiatry. (2015) 6:179. 10.3389/fpsyt.2015.00179 26779042 PMC4700272

[79] Steffens D Garrett M Soldano K McQuoid D Ashley-Koch A Potter G. Genome-wide screen to identify genetic loci associated with cognitive decline in late-life depression. Int Psychogeriatr. (2020) 1–9. 10.1017/S1041610220001143 32641180 PMC7794099

[80] Blanco-Sánchez B Clément A Fierro J Stednitz S Phillips J Wegner J. Grxcr1 promotes hair bundle development by destabilizing the physical interaction between harmonin and sans usher syndrome proteins. Cell Rep. (2018) 25:1281–91. 10.1016/j.celrep.2018.10.005 30380418 PMC6284068

[81] Wang T Han W Wang B Jiang Q Solberg-Woods L Palmer A. Propensity for social interaction predicts nicotine-reinforced behaviors in outbred rats. Genes Brain Behav. (2014) 13:202–12. 10.1111/gbb.12112 24289793 PMC3934210

[82] Temmingh H Stein D. Anxiety in patients with schizophrenia: epidemiology and management. CNS Drugs. (2015) 29:819–32. 10.1007/s40263-015-0282-7 26482261

[83] Howells F Kingdon D Baldwin D. Current and potential pharmacological and psychosocial interventions for anxiety symptoms and disorders in patients with schizophrenia: structured review. Hum Psychopharmacol. (2017) 32. 10.1002/hup.2628 28812313

[84] Larsen R Proue A Scott E Christiansen M Nakagawa Y. The thalamus regulates retinoic acid signaling and development of parvalbumin interneurons in postnatal mouse prefrontal cortex. eNeuro. 6. 10.1523/ENEURO.0018-19.2019 30868103 PMC6385081

[85] Samamé C. Social cognition throughout the three phases of bipolar disorder: a state-of-the-art overview. Psychiatry Res. (2013) 210:1275–86. 10.1016/j.psychres.2013.08.012 24075306

[86] Green M Horan W Lee J. Social cognition in schizophrenia. Nat Rev Neurosci. (2015) 16:620–31. 10.1038/nrn4005 26373471

[87] Kisko T Braun M Michels S Witt S Rietschel M Culmsee C. Cacna1c haploinsufficiency leads to pro-social 50-kHz ultrasonic communication deficits in rats. Dis Model Mech. (2018) 11. 10.1242/dmm.034116 29739816 PMC6031367

[88] Wöhr M Kisko T Schwarting R. Social behavior and ultrasonic vocalizations in a genetic rat model haploinsufficient for the cross-disorder risk gene cacna1c. Brain Sci. (2021) 11:724. 10.3390/brainsci11060724 34072335 PMC8229447

[89] Terrillion C Francis T Puche A Lobo M Gould T. Decreased nucleus accumbens expression of psychiatric disorder risk gene cacna1c promotes susceptibility to social stress. Int J Neuropsychopharmacol. (2017) 20:428–33. 10.1093/ijnp/pyw112 28165117 PMC5417061

[90] Kabir Z Che A Fischer D Rice R Rizzo B Byrne M. Rescue of impaired sociability and anxiety-like behavior in adult cacna1c-deficient mice by pharmacologically targeting eIF2alpha. Mol Psychiatry. (2017) 22:1096–109. 10.1038/mp.2017.124 28584287 PMC5863913

[91] Sittig L Carbonetto P Engel K Krauss K Barrios-Camacho C Palmer A. Genetic background limits generalizability of genotype-phenotype relationships. Neuron. (2016) 91:1253–9. 10.1016/j.neuron.2016.08.013 27618673 PMC5033712

[92] Arias-Hervert E Xu N Njus M Murphy G Hou Y Williams J. Actions of Rab27B-GTPase on mammalian central excitatory synaptic transmission. Physiol Rep. (2020) 8:e14428. 10.14814/phy2.14428 32358861 PMC7195558

[93] Shen Y Gu Y Su W Zhong J Jin Z Gu X. Rab27b is involved in lysosomal exocytosis and proteolipid protein trafficking in oligodendrocytes. Neurosci Bull. (2016) 32:331–40. 10.1007/s12264-016-0045-6 27325508 PMC5563785

[94] Kleene R Cassens C Bähring R Theis T Xiao M Dityatev A. Functional consequences of the interactions among the neural cell adhesion molecule NCAM, the receptor tyrosine kinase TrkB, and the inwardly rectifying K+ channel KIR3.3. J Biol Chem. (2010) 285:28968–79. 10.1074/jbc.M110.114876 20610389 PMC2937924

[95] Firestein B Firestein B Brenman J Aoki C Sanchez-Perez A El-Husseini A. Cypin: a cytosolic regulator of PSD-95 postsynaptic targeting. Neuron. (1999) 24:659–72. 10.1016/S0896-6273(00)81120-4 10595517

[96] Nicod J Davies R Cai N Hassett C Goodstadt L Cosgrove C. Genome-wide association of multiple complex traits in outbred mice by ultra-low-coverage sequencing. Nat Genet. (2016) 48:912–8. 10.1038/ng.3595 27376238 PMC4966644

[97] Ludin N Orts-Sebastian A Cheeseman J Chong J Merry A Cumin D. General anaesthesia shifts the murine circadian clock in a time-dependant fashion. Clocks Sleep. (2021) 3:87–97. 10.3390/clockssleep3010006 33530488 PMC7930986

[98] Schuch J Genro J Bastos C Ghisleni G Tovo-Rodrigues L. The role of CLOCK gene in psychiatric disorders: evidence from human and animal research. Am J Med Genet B Neuropsychiatr Genet. (2018) 177:181–98. 10.1002/ajmg.b.32599 28902457

[99] Smoller J Andreassen O Edenberg H Faraone S Glatt S Kendler K. Psychiatric genetics and the structure of psychopathology. Mol Psychiatry. (2019) 24:409–20. 10.1038/s41380-017-0010-4 29317742 PMC6684352

[100] Jones H Stergiakouli E Tansey K Hubbard L Heron J Cannon M. Phenotypic Manifestation of Genetic Risk for Schizophrenia During Adolescence in the General Population. JAMA Psychiatry. (2016) 73:221–8. 10.1001/jamapsychiatry.2015.3058 26818099 PMC5024747

[101] Cross-Disorder Group of the Psychiatric Genomics Consortium. Identification of risk loci with shared effects on five major psychiatric disorders: a genome-wide analysis. Lancet. (2013) 381:1371–9. 10.1016/S0140-6736(12)62129-1 23453885 PMC3714010

[102] Hatzimanolis A Bhatnagar P Moes A Wang R Roussos P Bitsios P. Common genetic variation and schizophrenia polygenic risk influence neurocognitive performance in young adulthood. Am J Med Genet B Neuropsychiatr Genet. (2015) 1688:392–401. 10.1002/ajmg.b.32323 25963331 PMC5008149

[103] Zhu J Chen Z Tian J Meng Z Ju M Wu G. miR-34b attenuates trauma-induced anxiety-like behavior by targeting CRHR1. Int J Mol Med. (2017) 40:90–100. 10.3892/ijmm.2017.2981 28498394 PMC5466391

[104] Fan J Wang X Hao K Yuan Y Chen X Du J. Upregulation of PVN CRHR1 by gestational intermittent hypoxia selectively triggers a male-specific anxiogenic effect in rat offspring. Horm Behav. (2013) 63:25–31. 10.1016/j.yhbeh.2012.11.005 23164543

[105] Wang X Labermaier C Holsboer F Wurst W Deussing J Müller M. Early-life stress-induced anxiety-related behavior in adult mice partially requires forebrain corticotropin-releasing hormone receptor 1. Eur J Neurosci. (2012) 36:2360–7. 10.1111/j.1460-9568.2012.08148.x 22672268

[106] Sey N Hu B Mah W Fauni H McAfee J Rajarajan P. A computational tool (H-MAGMA) for improved prediction of brain-disorder risk genes by incorporating brain chromatin interaction profiles. Nat Neurosci. (2020) 23:583–93. 10.1038/s41593-020-0603-0 32152537 PMC7131892

[107] Watanabe K Taskesen E van Bochoven A Posthuma D. Functional mapping and annotation of genetic associations with FUMA. Nat Commun. (2017) 8:1826. 10.1038/s41467-017-01261-5 29184056 PMC5705698

[108] King C Palmer A Woods L Hawk L Richards J Meyer P. Premature responding is associated with approach to a food cue in male and female heterogeneous stock rats. Psychopharmacology (Berl). (2016) 233:2593–605. 10.1007/s00213-016-4306-x 27146401 PMC5025873

[109] King C Tripi J Hughson A Horvath A Lamparelli A Holl K. Sensitivity to food and cocaine cues are independent traits in a large sample of heterogeneous stock rats. Sci Rep. (2021) 11:2223. 10.1038/s41598-020-80798-w 33500444 PMC7838206

[110] Sedighim S Carrette L Venniro M Shaham Y de Guglielmo G George O. Individual differences in addiction-like behaviors and choice between cocaine versus food in Heterogeneous Stock rats. Psychopharmacology (Berl). (2021) 238:3423–33. 10.1101/2021.07.21.453270 34415376 PMC8889911

[111] Carrette L de Guglielmo G Kallupi M Maturin L Brennan M Boomhower B. The cocaine and oxycodone biobanks, two repositories from genetically diverse and behaviorally characterized rats for the study of addiction. eNeuro. (2021) 8:ENEURO.0033-21.2021. 10.1523/ENEURO.0033-21.2021 33875455 PMC8213442

