# Supplementary material for: Genome-Wide Association Study on Three Behaviors Tested in an Open Field in Heterogeneous Stock Rats Identifies Multiple Loci Implicated in Psychiatric Disorders
Source: Front Psychiatry. 2022 Feb 14;13:790566. doi: 10.3389/fpsyt.2022.790566 (PMC8882588; doi:10.3389/fpsyt.2022.790566)
Supplement: Supplementary file 1 [file Data_Sheet_1.pdf]

Table S1. Sex differences of OFT, NOIT and SIT (Mean ± SD)

Test	Trait	Female	Male	p-value	Cohen's d
OFT	Duration in center zone	65 ± 51.4	55.7 ± 43.5	0.0006	0.19
	Frequency of entering center zone	22.7 ± 14	19.9 ± 13.7	0.0003	0.21
	Latency of entering center zone	250.8 ± 301.3	271.6 ± 305.1	0.23	0.07
	Mean distance to center zone	36.3 ± 3.9	37.1 ± 3.9	0.0002	0.21
	Total distance to center zone	1930273 ± 224949	1976133 ± 229112	0.0004	0.20
	Total travel distance	16578 ± 4372	15762 ± 4272	0.0009	0.19
NOIT	Duration in center zone	283.9 ± 151.7	268.1 ± 160.7	0.08	0.10
	Frequency of entering center zone	40.4 ± 19.3	40.2 ± 20.6	0.92	0.01
	Latency of entering center zone	88.4 ± 123.8	108.3 ± 148	0.01	0.15
	Mean distance to center zone	24.6 ± 7.2	26.6 ± 7.3	<0.0001	0.27
	Total distance to center zone	437507 ± 127709	473355 ± 130656	<0.0001	0.28
	Total travel distance	8611 ± 2113	8217 ± 2226	0.001	0.18
SIT	Duration in object zone	141.2 ± 77.4	163.1 ± 86.9	<0.0001	0.27
	Duration in social zone	454.3 ± 208.8	392.4 ± 187.5	<0.0001	0.31
	Frequency of entering object zone	44.9 ± 20.8	50 ± 25.1	<0.0001	0.22
	Frequency of entering social zone	85.2 ± 37.2	87 ± 32.7	0.35	0.05
	Latency of entering object zone	35.9 ± 51.2	37 ± 68.5	0.73	0.02
	Latency of entering social zone	5.2 ± 22.8	7.4 ± 45	0.28	0.06
	Mean distance to object zone	22.6 ± 6.7	23.6 ± 6.8	0.01	0.14
	Mean distance to social zone	12.3 ± 4.9	13.7 ± 4.5	<0.0001	0.30
	Total distance to object zone	400955 ± 117623	420714 ± 121683	0.003	0.17
	Total distance to social zone	218013 ± 87249	244171 ± 80511	<0.0001	0.31
	Total travel distance	9819 ± 2239	9841 ± 2312	0.86	0.01

Table S2. Strain distribution pattern of nearby QTL

Top SNP	AF	SE	Beta	Allele1	Allele2	ACI	BN	BUF	F344	M520	MR	WN	WKY
chr4:150582701	0.362	0.042	-0.203	C	A	AA	CC	AA	AA	AA	AA	AA	AA
chr4:151128675	0.512	0.040	-0.192	C	A	CC	CC	CC	CC	CC	CC	CC	AA
chr6:119975012	0.696	0.048	-0.229	C	T	CC	CC	CC	CC	TT	CC	CC	CC
chr6:120117521	0.312	0.046	-0.216	T	G	GG	TT	00	GG	TT	GG	GG	GG
chr19:20666789	0.946	0.088	-0.458	G	C	GG	GG	GG	GG	CC	GG	GG	CC
chr19:20667417	0.946	0.088	-0.452	A	G	AA	AA	AA	AA	AA	AA	AA	GG

Table S3. Pleiotropic effects.

Top SNP	Trait	$-log_{10}P$
chr1:144080083	NOIT distance to center, total	5.969
	NOIT center duration	4.633
	NOIT distance to center, mean	5.143
chr4:58009499	OFT distance to center, total	7.254
	OFT distance to center, mean	7.469
	OFT total travel distance	4.191
chr4:112234344	NOIT center duration	6.028
	NOIT distance to center, mean	6.598
	NOIT distance to center, total	5.975
chr4:118013062	OFT center frequency	5.777
	OFT distance to center, total	6.099
	OFT center duration	5.373
	OFT distance to center, mean	5.001
chr4:150582701	SIT distance to social zone, mean	5.884
	SIT distance to social zone, total	5.788
	SIT in social zone duration	4.324
chr4:151128675	SIT in social zone duration	5.820
	SIT distance to social zone mean	4.351
	SIT distance to social zone, total	4.366
chr4:156801420	NOIT distance to center, total	5.622
	NOIT distance to center, mean	4.543
chr6:119975012	NOIT distance to center, mean	5.692
	NOIT center frequency	4.275
	NOIT distance to center, total	5.542
	NOIT total travel distance	5.270
chr6:120117521	NOIT total travel distance	5.640
	NOIT center frequency	4.330
	NOIT distance to center, mean	5.021
	NOIT distance to center, total	4.961
chr11:33359859	OFT total travel distance	8.268
	OFT center duration	4.125
	OFT center frequency	4.895
chr14:44904830	OFT distance to center, total	5.741
	OFT center frequency	4.189
	OFT distance to center, mean	5.096
chr14:41727329	SIT total travel distance	5.627
	NOIT total travel distance	4.291
chr19:20666789	SIT distance to object zone, mean	6.746
	SIT distance to object zone, total	6.613
chr19:20667417	SIT distance to object zone, total	6.619
	SIT distance to object zone, mean	6.732
chr19:55339863	SIT distance to social zone, mean	6.661
	SIT distance to social zone, total	6.643

This is a provisional file, not the final typeset article

Table S4. Putatively causal coding variants, HIGH impact

Chr	Location	ref	alt	effect	gene	cDNA position	SNP change	Amino acid change	r2 with trait change	dprime	phenotype
4	156219233	G	T	stop gained	Clec4n	505	c.505G>T	p.Glu169*	0.801402	1	NOIT
4	156219233	G	T	stop gained	Clec4n	415	c.415G>T	p.Glu139*	0.801402	1	NOIT
4	156219233	G	T	stop gained	Clec4n	403	c.403G>T	p.Glu135*	0.801402	1	NOIT
4	156219240	G	A	stop gained	Clec4n	512	c.512G>A	p.Trp171*	0.801402	1	NOIT
4	156219240	G	A	stop gained	Clec4n	422	c.422G>A	p.Trp141*	0.801402	1	NOIT
4	156219240	G	A	stop gained	Clec4n	410	c.410G>A	p.Trp137*	0.801402	1	NOIT
13	90703234	T	C	stop lost	Kcnj9	1618	c.1236A>G	p.Ter412Trpext*?	0.99127	0.997078	SIT
19	21049487	C	T	stop gained	Rps15a	343	c.343C>T	p.Arg115*	0.700642	0.936371	SIT

Table S5. Hot spot on rat Chr1 144Mb loci has 17 genes associated psychiatric conditions in human

Chr (Human)	Location	SNP	Gene/Region	P-val	P-val note	Disease/Trait	Mapped trait	PMID
15	82558175	rs17507216	CPEB1	4E-10		Cognitive ability, years of educational attainment or schizophrenia (pleiotropy)	schizophrenia, intelligence, self reported educational attainment	31374203
15	82585958	rs783540	CPEB1	2E-08		Schizophrenia	schizophrenia	31740837
15	82585958	rs783540	CPEB1	3E-08		Schizophrenia	schizophrenia	28991256
15	82585958	rs783540	CPEB1	5E-08	(EA)	Schizophrenia	schizophrenia	30285260
15	82585958	rs783540	CPEB1	7E-08		Schizophrenia	schizophrenia	26198764

Continuation of Table

15	82585958	rs783540	CPEB1	9E-09		Schizophrenia	schizophrenia	30285260
15	83591790	rs141308780	SH3GL3	3E-06		Schizophrenia	schizophrenia	26198764
15	83845538	rs7162542	ADAMTSL3	1E-14	(men)	Waist circumference adjusted for BMI (adjusted for smoking behaviour)	smoking behavior	28443625
15	83845538	rs7162542	ADAMTSL3	2E-12	(women)	Waist circumference adjusted for BMI (adjusted for smoking behaviour)	smoking behavior	28443625
15	83845538	rs7162542	ADAMTSL3	2E-16	(men)	Waist circumference adjusted for BMI (joint analysis main effects and smoking interaction)	smoking behavior	28443625
15	83845538	rs7162542	ADAMTSL3	2E-23		Waist circumference adjusted for BMI (adjusted for smoking behaviour)	smoking behavior	28443625
15	83845538	rs7162542	ADAMTSL3	5E-12	(women)	Waist circumference adjusted for BMI (joint analysis main effects and smoking interaction)	smoking behavior	28443625
15	83845538	rs7162542	ADAMTSL3	5E-25		Waist circumference adjusted for BMI (joint analysis main effects and smoking interaction)	smoking behavior	28443625
15	84037709	rs950169	ADAMTSL3	1E-10	(EA)	Schizophrenia	schizophrenia	30285260
15	84037709	rs950169	ADAMTSL3	2E-11		Schizophrenia	schizophrenia	25056061
15	84037709	rs950169	ADAMTSL3	2E-11		Schizophrenia	schizophrenia	30285260
15	84037709	rs950169	ADAMTSL3	3E-11		Schizophrenia	schizophrenia	26198764

Continuation of Table

15	84037709	rs950169	ADAMTSL3	5E-12	Schizophrenia	schizophrenia	28991256
15	84581411	rs11633534	LINC00933 - ZSCAN2	8E-14	Schizophrenia	schizophrenia	31740837
15	84582607	rs12902052	LINC00933 - ZSCAN2	7E-09	Bipolar disorder	bipolar disorder	33263727
15	84594463	rs11638445	LINC00933 - ZSCAN2	3E-10	Cognitive ability, years of educational attainment or schizophrenia (pleiotropy)	schizophrenia, intelligence, self reported educational attainment	31374203
15	84606344	rs748455	ZSCAN2	6E-13	Schizophrenia (MTAG)	schizophrenia	32606422
15	84610573	rs71395455	ZSCAN2	4E-08	Bipolar I disorder	bipolar I disorder	31043756
15	84610573	rs71395455	ZSCAN2	5E-14	Anorexia nervosa, attention-deficit/hyperactivity disorder, autism spectrum disorder, bipolar disorder, major depression, obsessive-compulsive disorder, schizophrenia, or Tourette syndrome (pleiotropy)	anorexia nervosa, obsessive-compulsive disorder, attention deficit hyperactivity disorder, Tourette syndrome, unipolar depression, schizophrenia, autism spectrum disorder, bipolar disorder	31835028
15	84664594	rs12908161	NMB - SEC11A	5E-10	Schizophrenia	schizophrenia	26198764

Continuation of Table

15	84664594	rs12908161	NMB - SEC11A	9E-10		Schizophrenia	schizophrenia	29483656
15	84676882	rs55945116	SEC11A	6E-07		Response to ketamine in bipolar disorder or major depression (antidepressant effects)	unipolar depression, depressive symptom measurement, response to ketamine, bipolar disorder	30552317
15	84680172	rs12440825	SEC11A	1E-06	(Japanese)	3-hydroxy-1-methylpropylmercapturic acid levels in smokers	smoking behavior, 3-hydroxy-1-methylpropylmercapturic acid measurement	26053186
15	84812610	rs35828350	ZNF592 - ALPK3	6E-11		Autism spectrum disorder or schizophrenia	schizophrenia, autism spectrum disorder	28540026
15	84833784	rs891288	ALPK3	6E-07	(Japanese)	3-hydroxy-1-methylpropylmercapturic acid levels in smokers	smoking behavior, 3-hydroxy-1-methylpropylmercapturic acid measurement	26053186
15	88180481	rs1104918	NTRK3	6E-06	(Modelling analysis)	Autism spectrum disorder, attention deficit-hyperactivity disorder, bipolar disorder, major depressive disorder, and schizophrenia (combined)	attention deficit hyperactivity disorder, unipolar depression, schizophrenia, autism spectrum disorder, bipolar disorder	23453885
15	88215343	rs146797905	NTRK3	4E-07		Schizophrenia	schizophrenia	26198764

Continuation of Table

15	88327931	rs62024303	NTRK3-AS1 - NA	3E-07	(Latino)	3-hydroxypropylmercapturic acid levels in smokers	smoking behavior, 3-hydroxypropylmercapturic acid measurement	26053186
15	88402647	rs28541419	NA - MRPL46	3E-08		Depression (broad)	unipolar depression	29662059
15	88871064	rs1879529	ACAN	1E-06	(women)	Waist circumference adjusted for BMI (joint analysis main effects and smoking interaction)	smoking behavior	28443625
15	88871064	rs1879529	ACAN	1E-07		Waist circumference adjusted for BMI (adjusted for smoking behaviour)	smoking behavior	28443625
15	88871064	rs1879529	ACAN	8E-08		Waist circumference adjusted for BMI (joint analysis main effects and smoking interaction)	smoking behavior	28443625
15	88871064	rs1879529	ACAN	9E-07	(women)	Waist circumference adjusted for BMI (adjusted for smoking behaviour)	smoking behavior	28443625
15	89318595	rs2307441	POLG	3E-06	(HAM-A)	Venlafaxine response in generalised anxiety disorder (responders vs non-responders after 24 weeks)	response to venlafaxine, generalized anxiety disorder	28437668
15	89356393	rs12595305	POLG-DT - MIR9-3HG	4E-06		Schizophrenia	schizophrenia	26198764
15	89357656	rs758129	POLG-DT - MIR9-3HG	3E-07		Schizophrenia	schizophrenia	30285260

Continuation of Table

15	89357656	rs758129	POLG-DT - MIR9-3HG	3E-08		Schizophrenia	schizophrenia	28991256
15	89357656	rs758129	POLG-DT - MIR9-3HG	6E-06	(EA)	Schizophrenia	schizophrenia	30285260
15	89357656	rs758129	POLG-DT - MIR9-3HG	8E-10		Schizophrenia	schizophrenia	31740837
15	89370401	rs176644	MIR9-3HG	3E-09		Smoking status (ever vs never smokers)	smoking behavior	30643258
15	89694587	rs8035597	WDR93	6E-07		Deliberate self-harm	Self-injurious behavior	32473944
15	89729839	rs12148067	WDR93	9E-07		Deliberate self-harm	Self-injurious behavior	32473944
15	90873320	rs17514846	FURIN	3E-12		Schizophrenia	schizophrenia	29483656
15	90873320	rs17514846	FURIN	6E-11	(EA)	Schizophrenia	schizophrenia	30285260
15	90875067	rs8032315	FURIN	2E-08		Autism spectrum disorder or schizophrenia	schizophrenia, autism spectrum disorder	28540026
15	90883330	rs4702	FURIN	1E-10		Autism spectrum disorder or schizophrenia	schizophrenia, autism spectrum disorder	28540026

Continuation of Table

15	90883330	rs4702	FURIN	2E-11		Anorexia nervosa, attention-deficit/hyperactivity disorder, autism spectrum disorder, bipolar disorder, major depression, obsessive-compulsive disorder, schizophrenia, or Tourette syndrome (pleiotropy)	anorexia nervosa, obsessive-compulsive disorder, attention deficit hyperactivity disorder, Tourette syndrome, unipolar depression, schizophrenia, autism spectrum disorder, bipolar disorder	31835028
15	90883330	rs4702	FURIN	2E-11		Schizophrenia	schizophrenia	31268507
15	90883330	rs4702	FURIN	2E-15		Schizophrenia	schizophrenia	31740837
15	90883330	rs4702	FURIN	3E-11		Cognitive ability, years of educational attainment or schizophrenia (pleiotropy)	schizophrenia, intelligence, self reported educational attainment	31374203
15	90883330	rs4702	FURIN	3E-12		Schizophrenia	schizophrenia	26198764
15	90883330	rs4702	FURIN	5E-11		Bipolar disorder (MTAG)	bipolar disorder	32606422
15	90883330	rs4702	FURIN	5E-12		Schizophrenia (MTAG)	schizophrenia	32606422
15	90883330	rs4702	FURIN	8E-14		Schizophrenia (MTAG)	schizophrenia	32606422
15	90883330	rs4702	FURIN	8E-14		Schizophrenia	schizophrenia	25056061

Continuation of Table

15	90885060	rs11539637	FES	4E-09	Schizophrenia	schizophrenia	30285260

All data obtained from GWAS catalog (**?**)

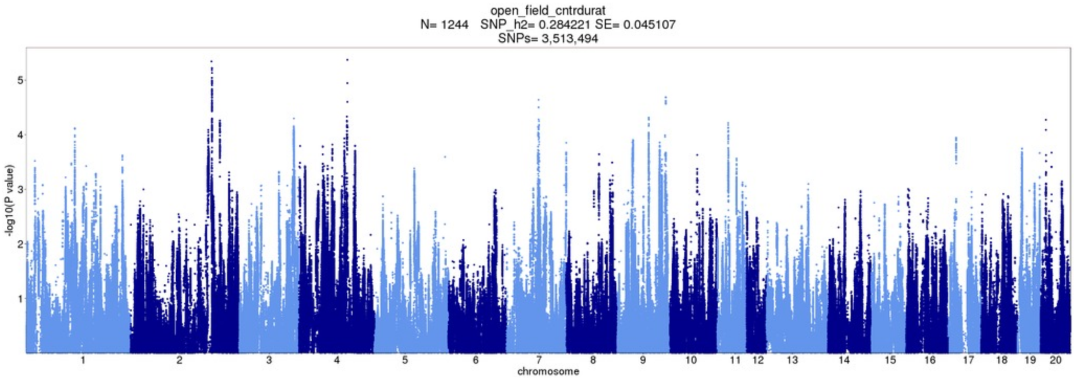

Figure S1. OFT GWAS: Duration in center zone, mean

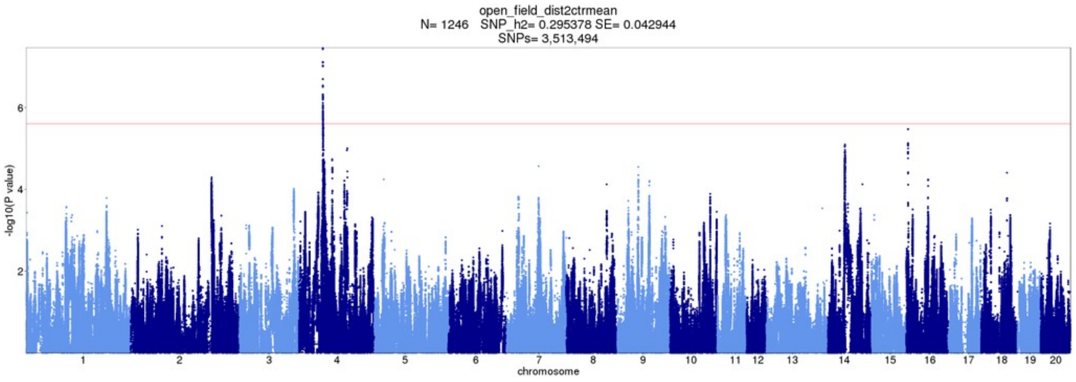

Figure S2. OFT GWAS: Mean distance to center zone

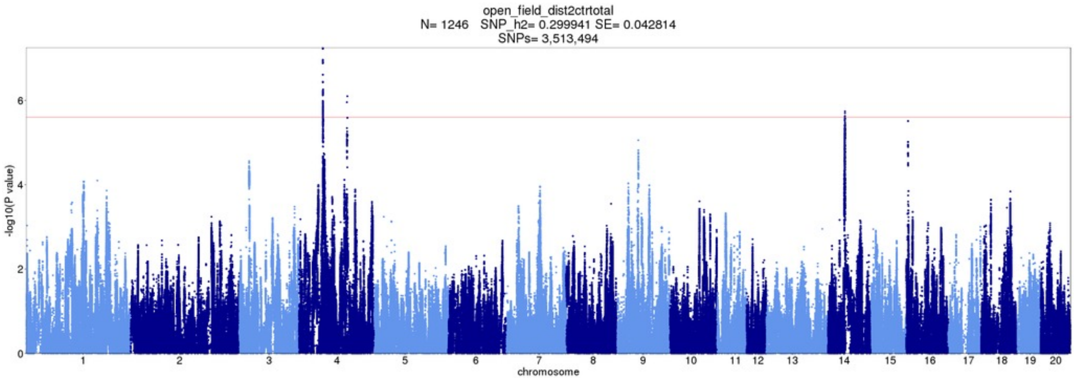

Figure S3. OFT GWAS: Total distance to center zone

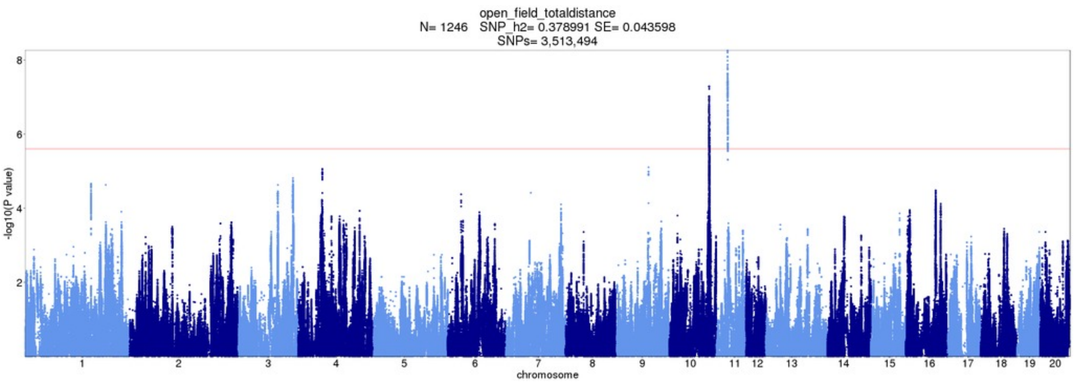

Figure S4. OFT GWAS: Total travel distance

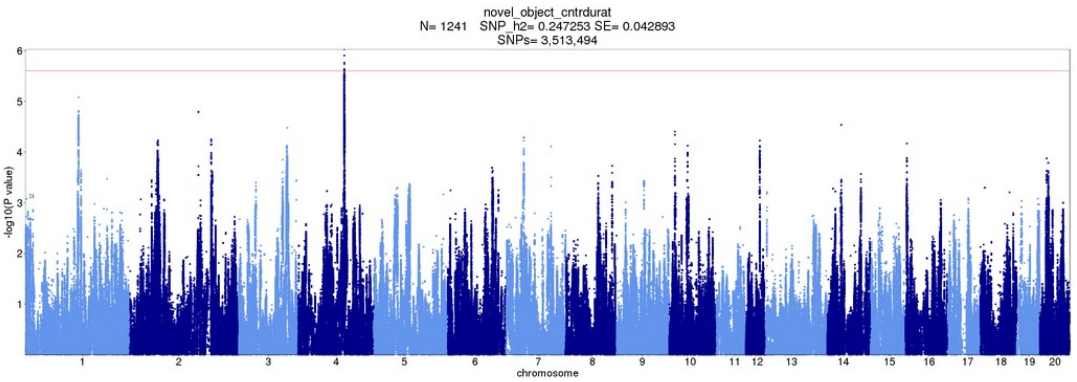

Figure S5. NOIT GWAS: Duration in center zone

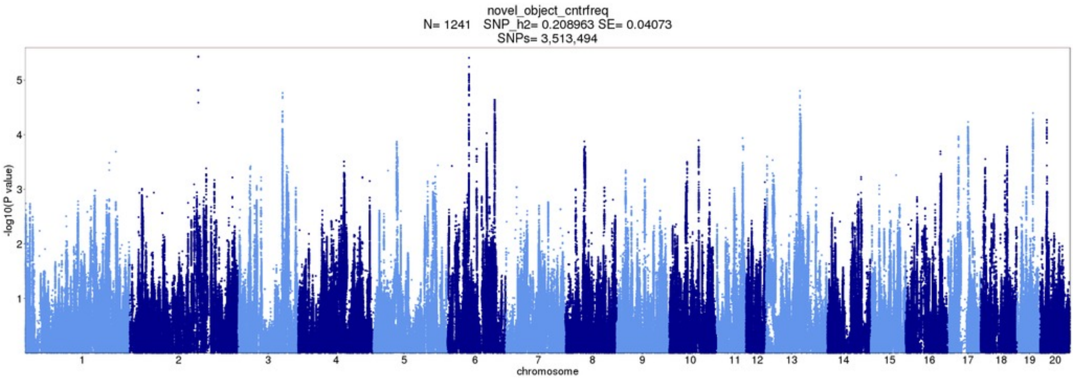

Figure S6. NOIT GWAS: Frequency of entering center zone

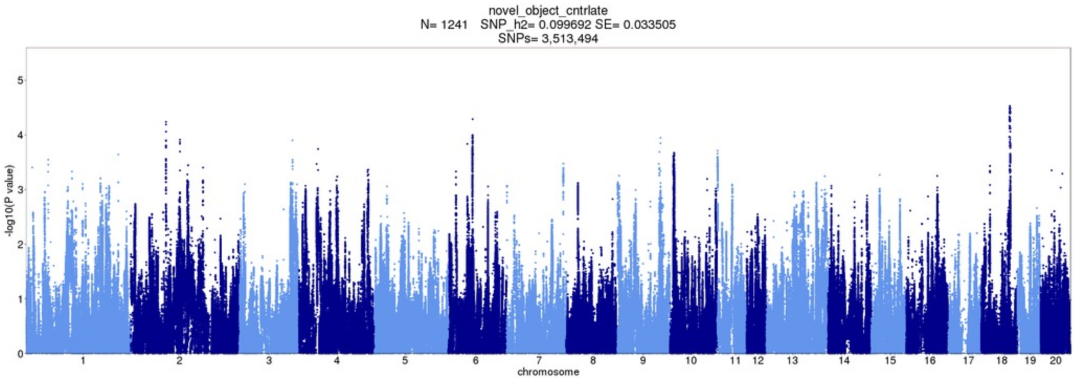

Figure S7. NOIT GWAS: Latency of entering center zone

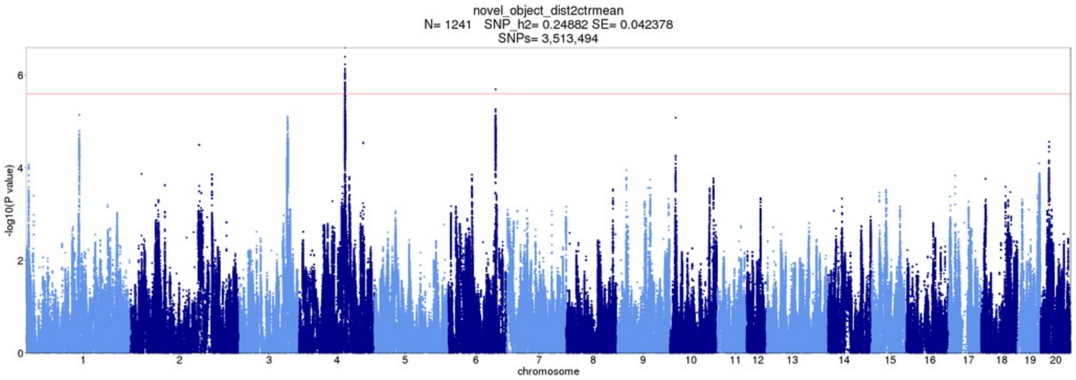

Figure S8. NOIT GWAS: Mean distance to center zone

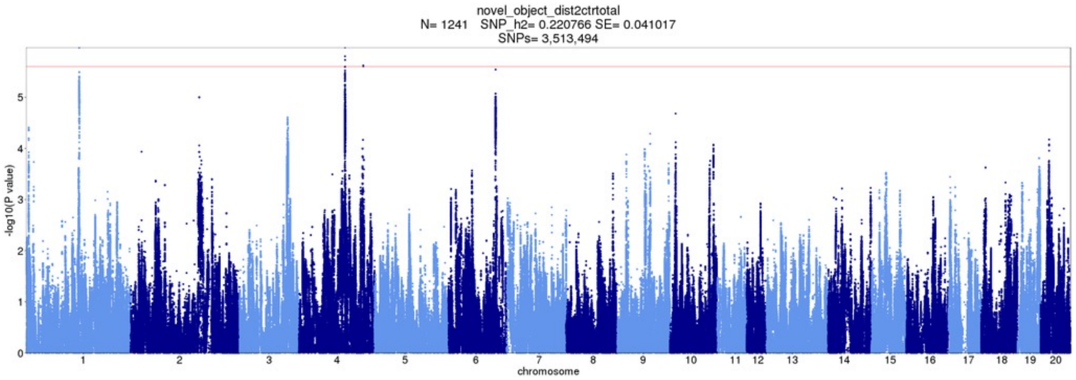

Figure S9. NOIT GWAS: Total distance to center zone

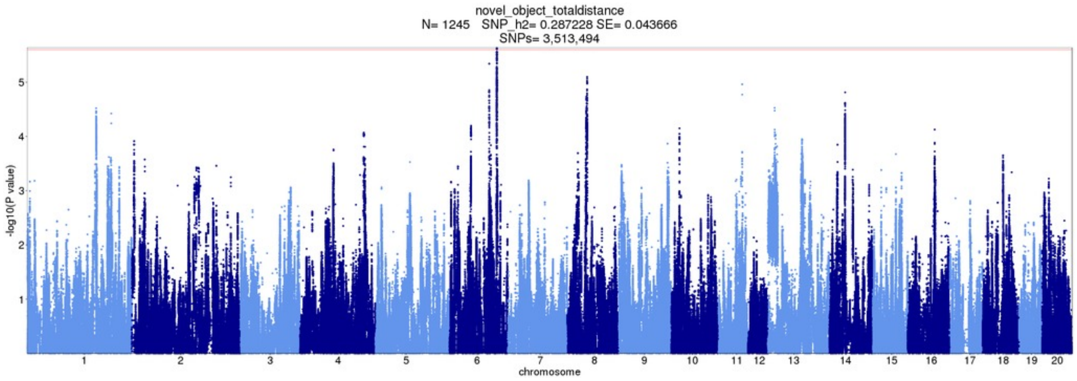

Figure S10. NOIT GWAS: Total travel distance

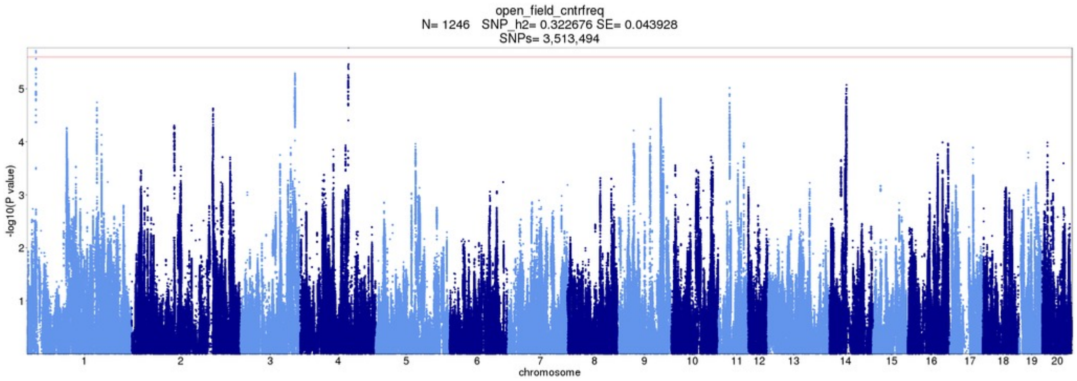

Figure S11. NOIT GWAS: Frequency of entering center zone, mean

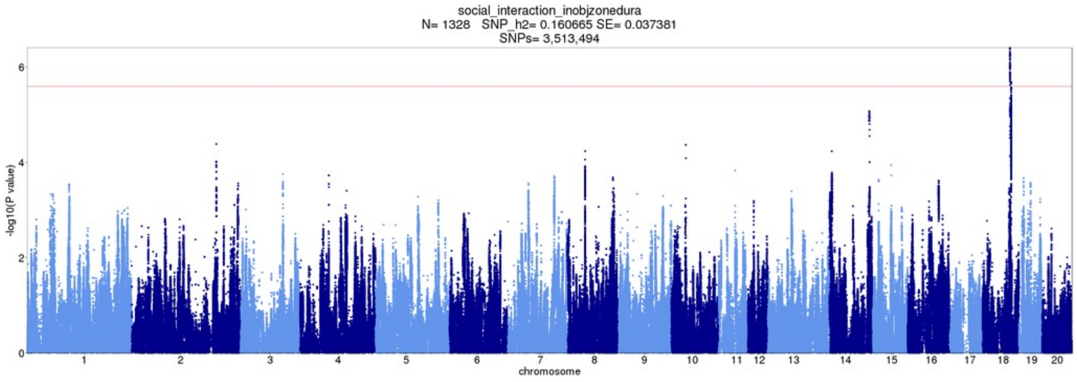

Figure S12. SIT GWAS: Duration in object zone

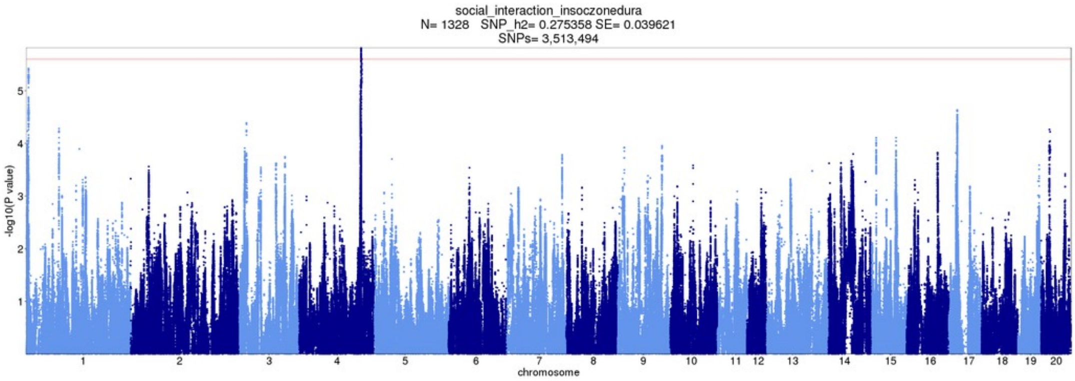

Figure S13. SIT GWAS: Duration in social zone

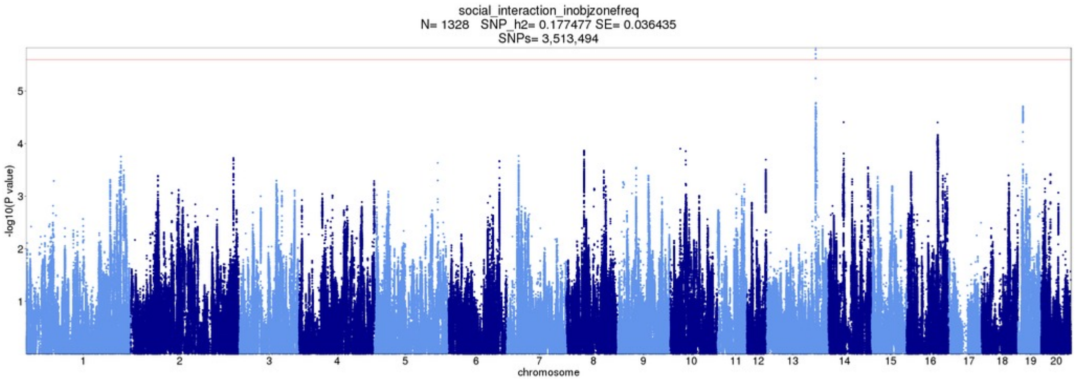

Figure S14. SIT GWAS: Frequency of entering object zone

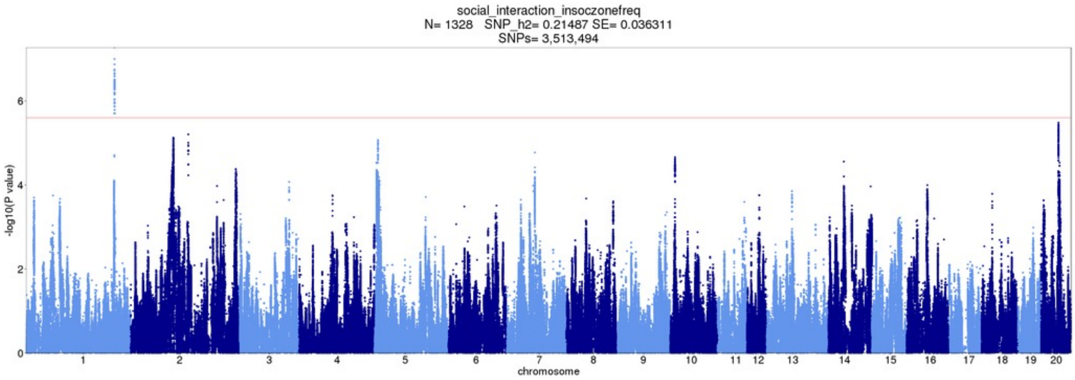

Figure S15. SIT GWAS: Frequency of entering social zone

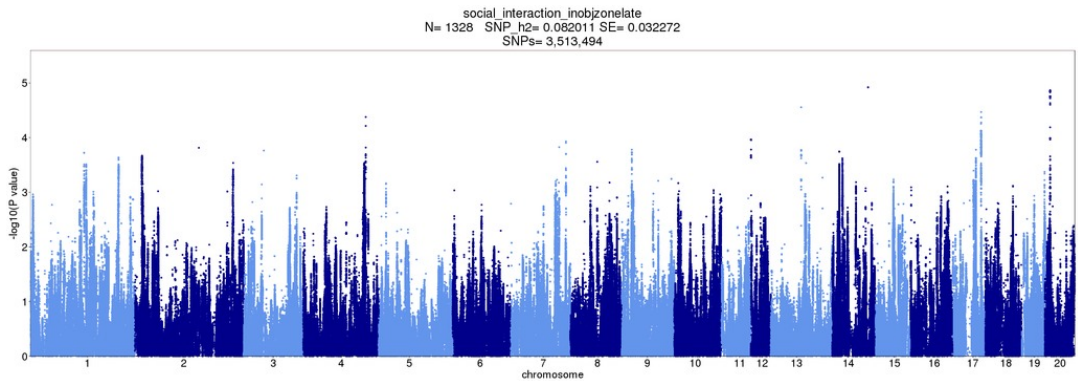

Figure S16. SIT GWAS: Latency of entering object zone

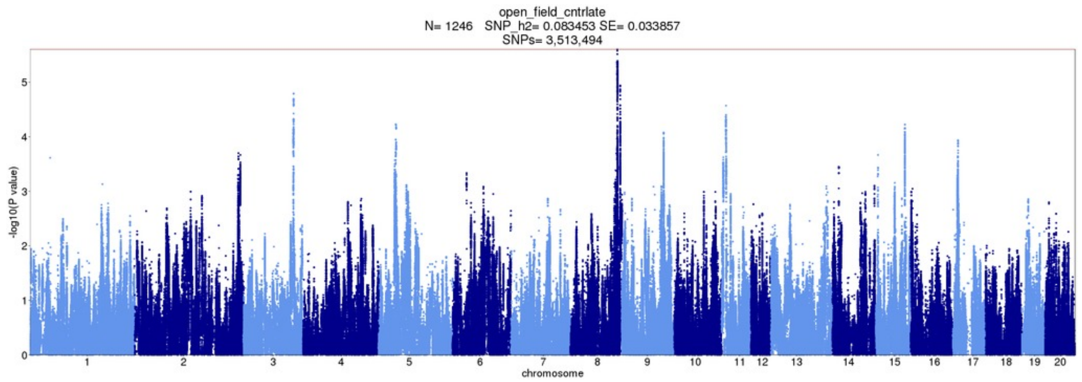

Figure S17. SIT GWAS: Latency of entering center zone, mean

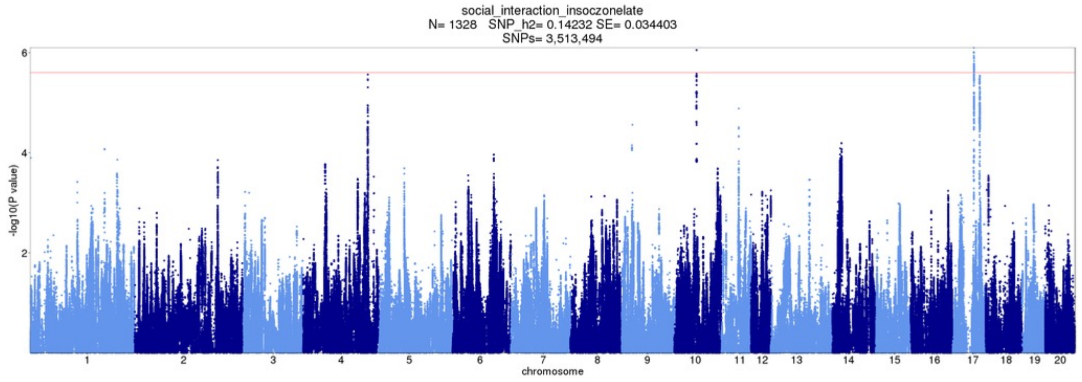

Figure S18. SIT GWAS: Latency of entering social zone

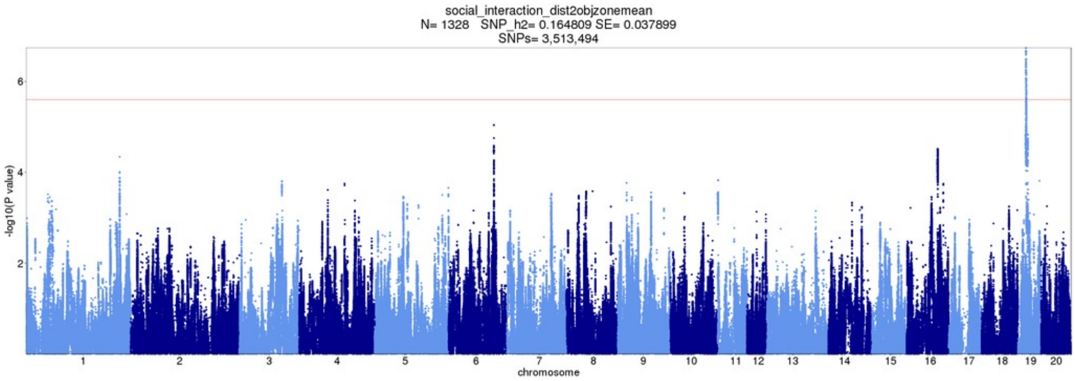

Figure S19. SIT GWAS: Mean distance to object zone

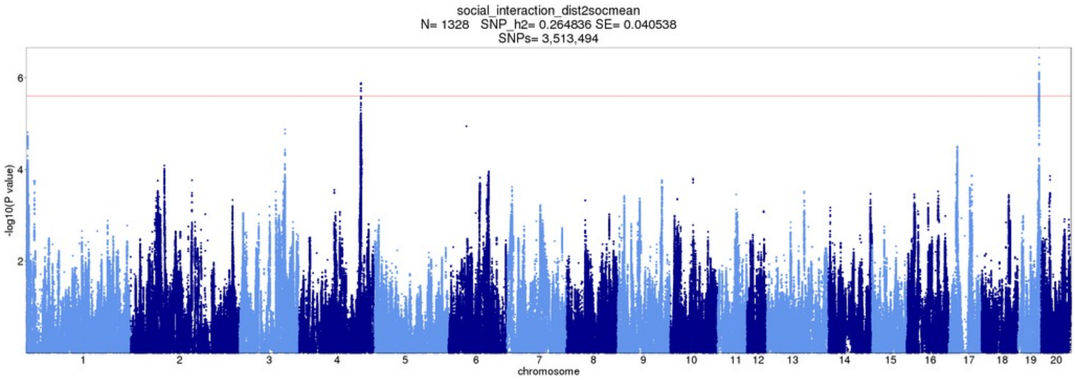

Figure S20. SIT GWAS: Mean distance to social zone

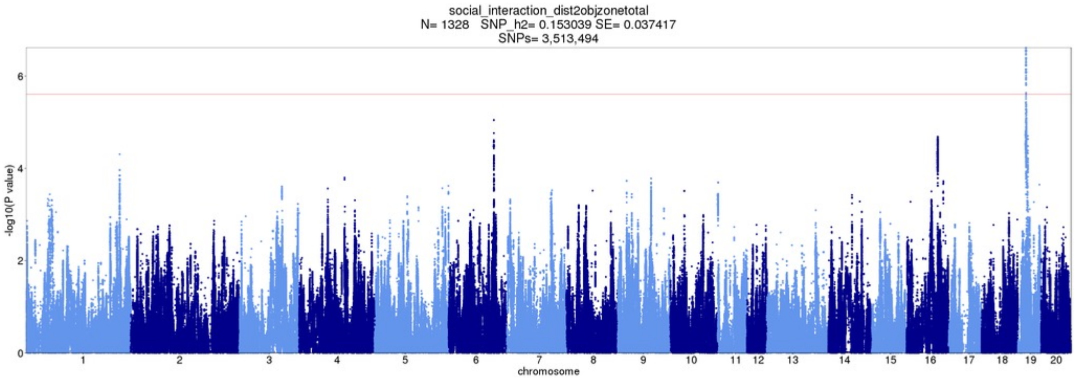

Figure S21. SIT GWAS: Total distance to object zone

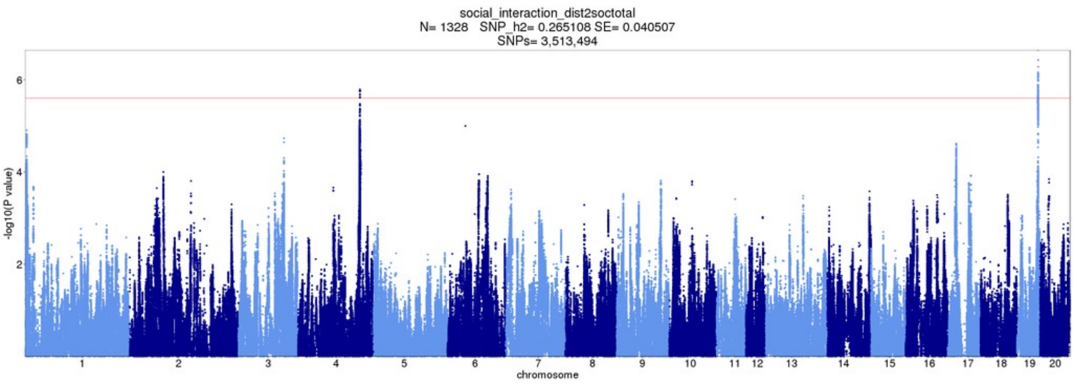

Figure S22. SIT GWAS: Total distance to social zone

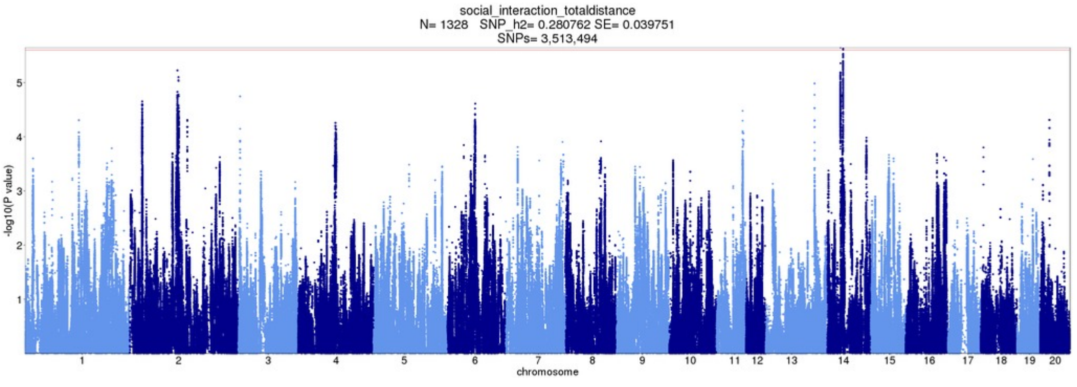

Figure S23. SIT GWAS: Total travel distance

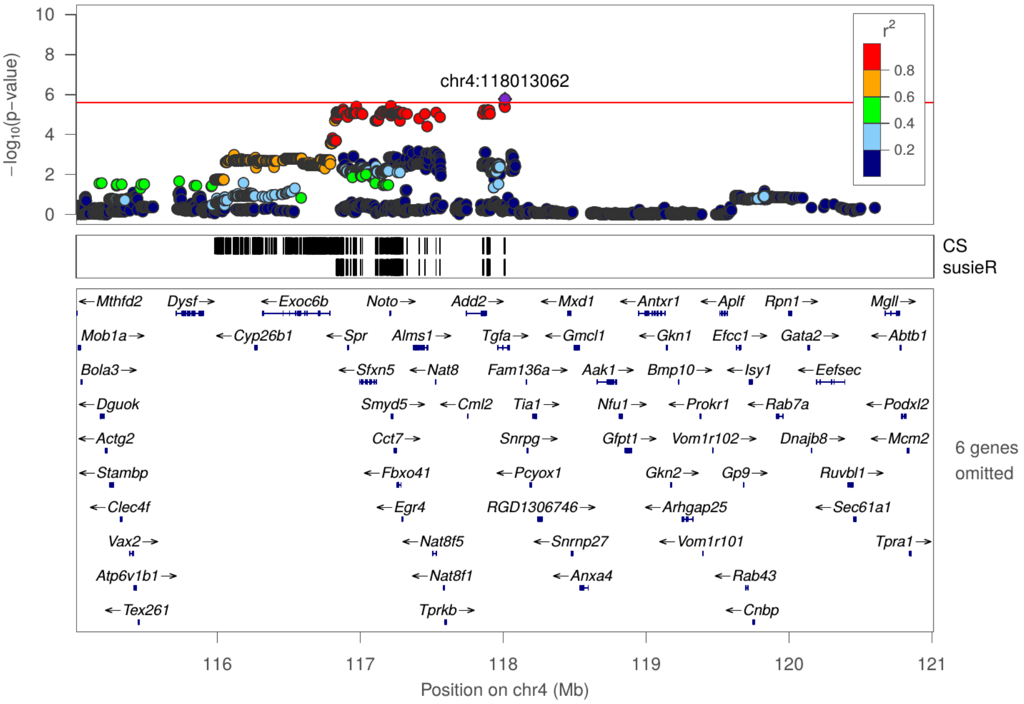

Figure S24. Regional association plot for OFT: Frequency of entering center zone at chr4:118013062

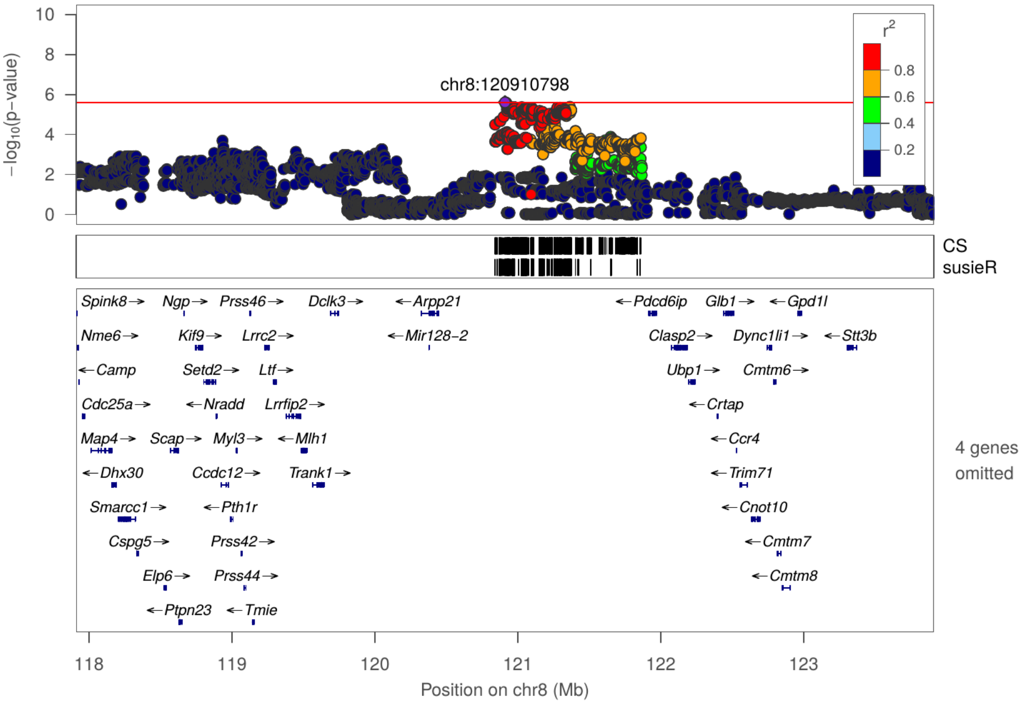

Figure S25. Regional association plot for OFT: Latency of entering center zone at chr8:120910798

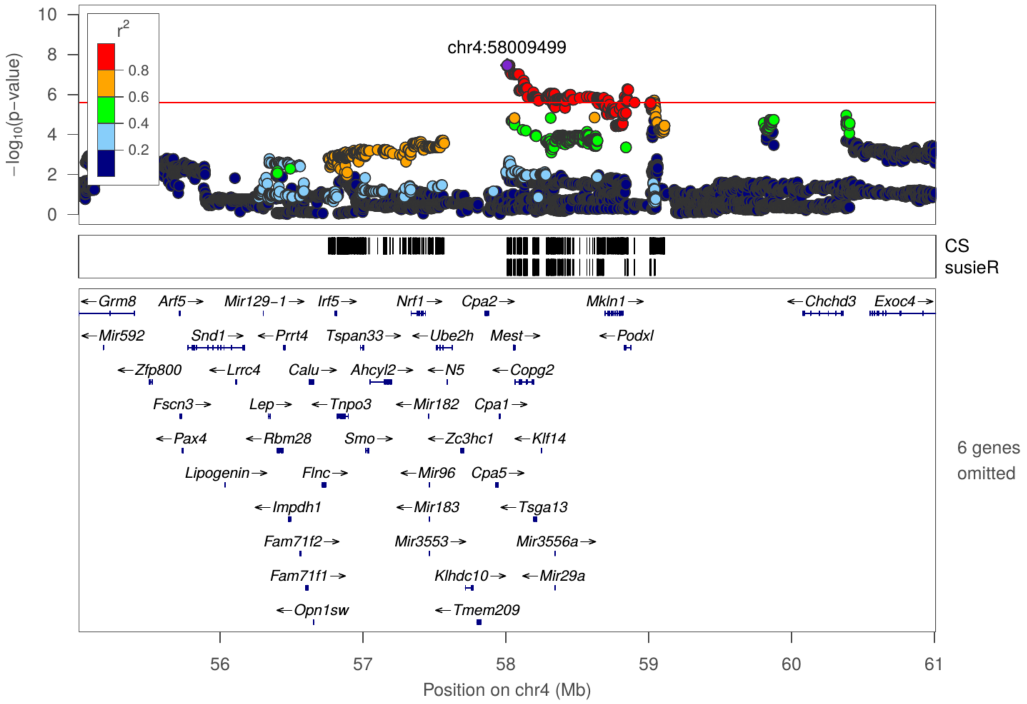

Figure S26. Regional association plot for OFT: Mean distance to center zone at chr4:58009499

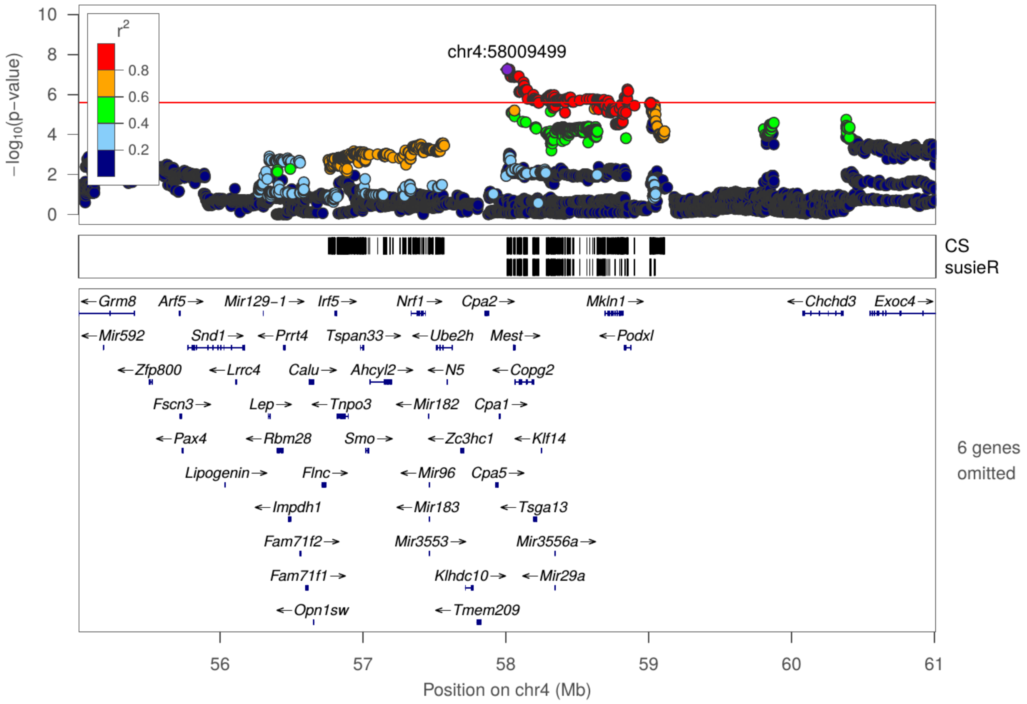

Figure S27. Regional association plot for OFT: Total distance to center zone at chr4:58009499

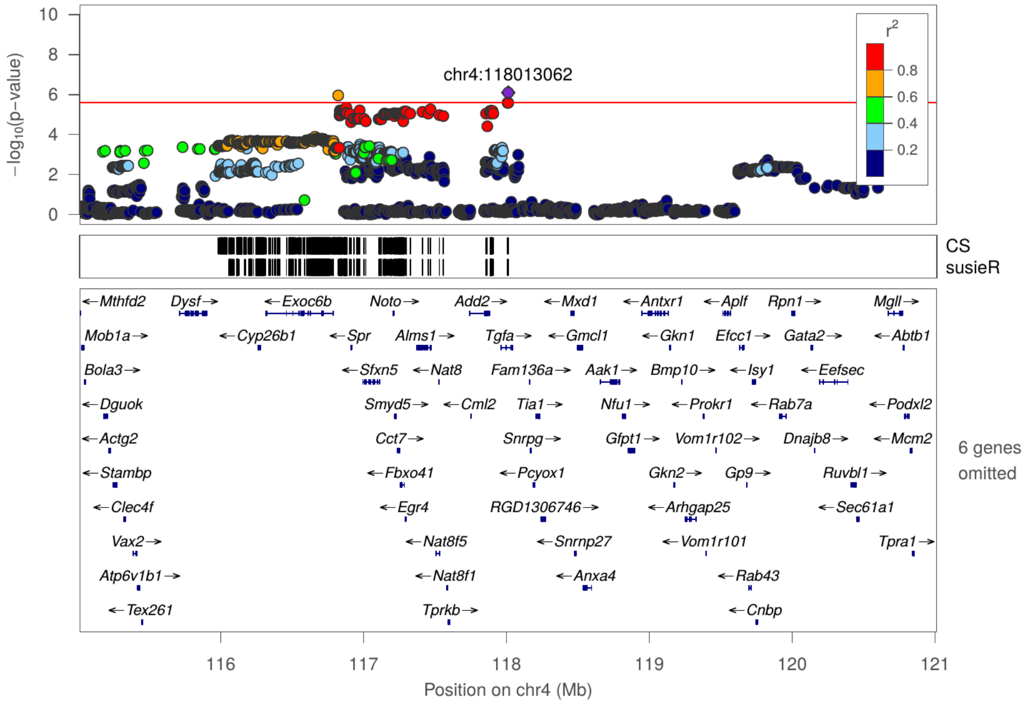

Figure S28. Regional association plot for OFT: Total distance to center zone at chr4:118013062

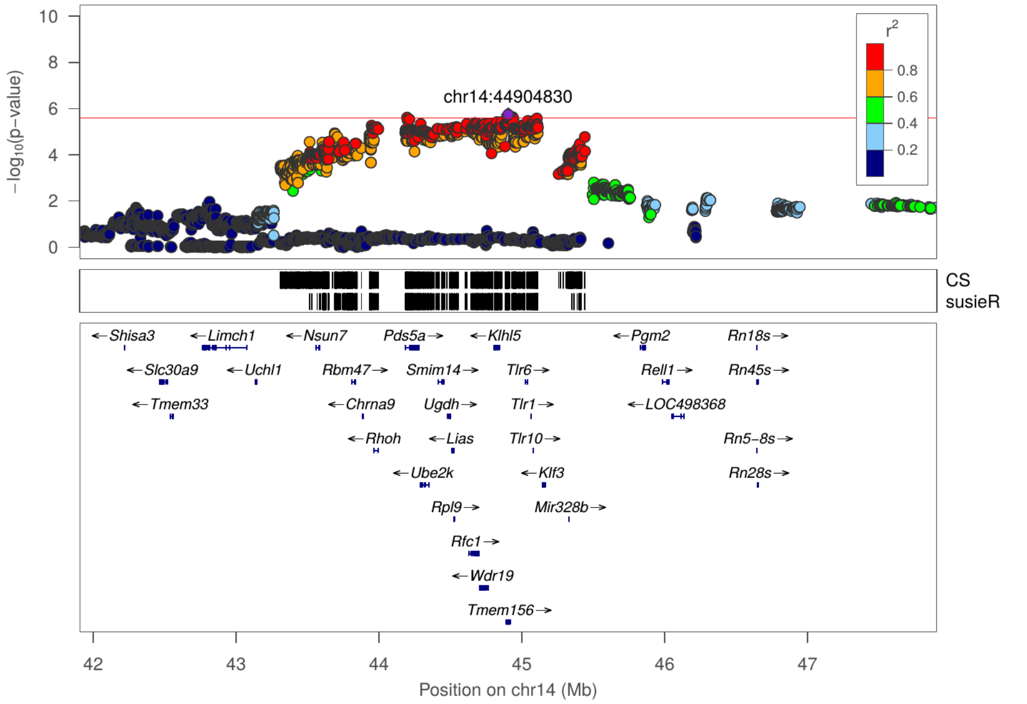

Figure S29. Regional association plot for OFT: Total distance to center zone at chr14:44904830

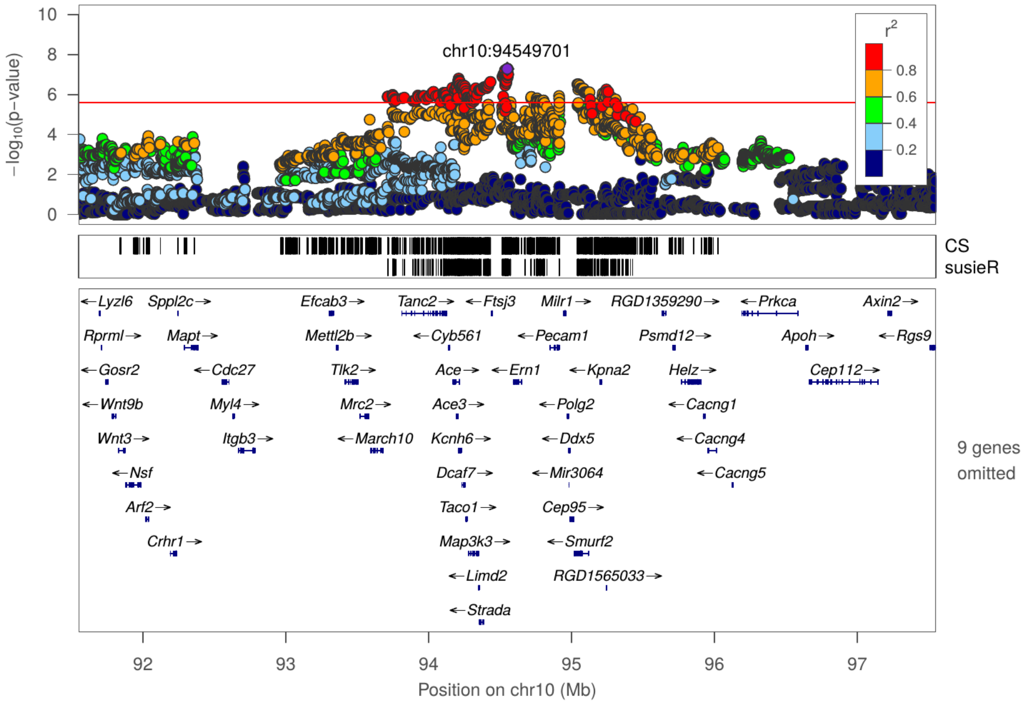

Figure S30. Regional association plot for OFT: Total travel distance at chr10:94549701

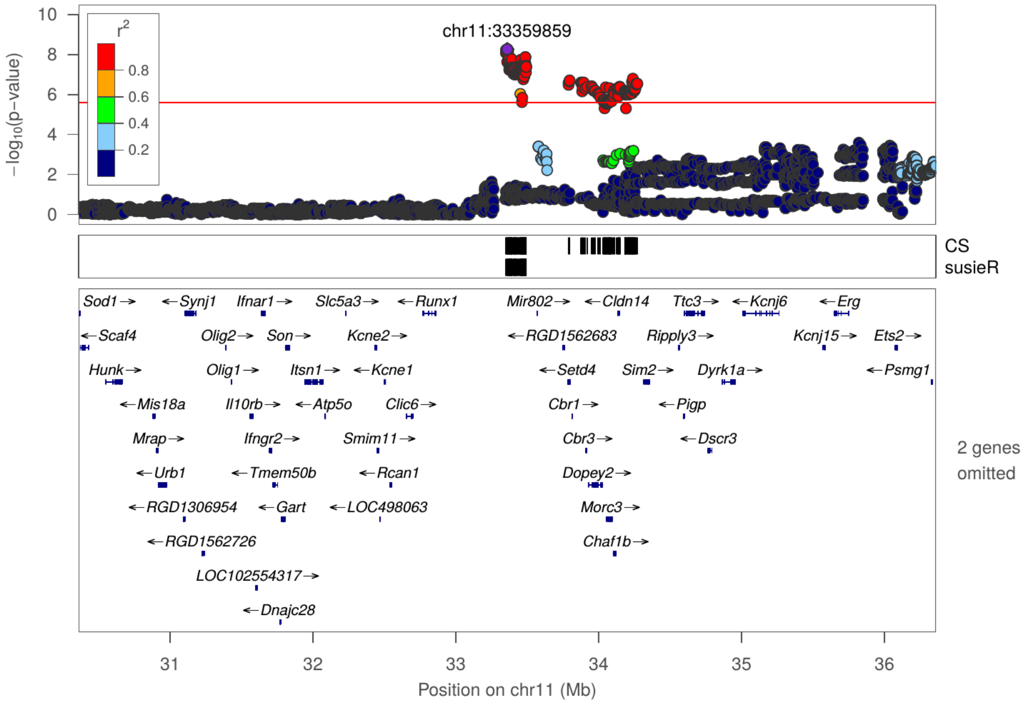

Figure S31. Regional association plot for OFT: Total travel distance at chr11:33359859

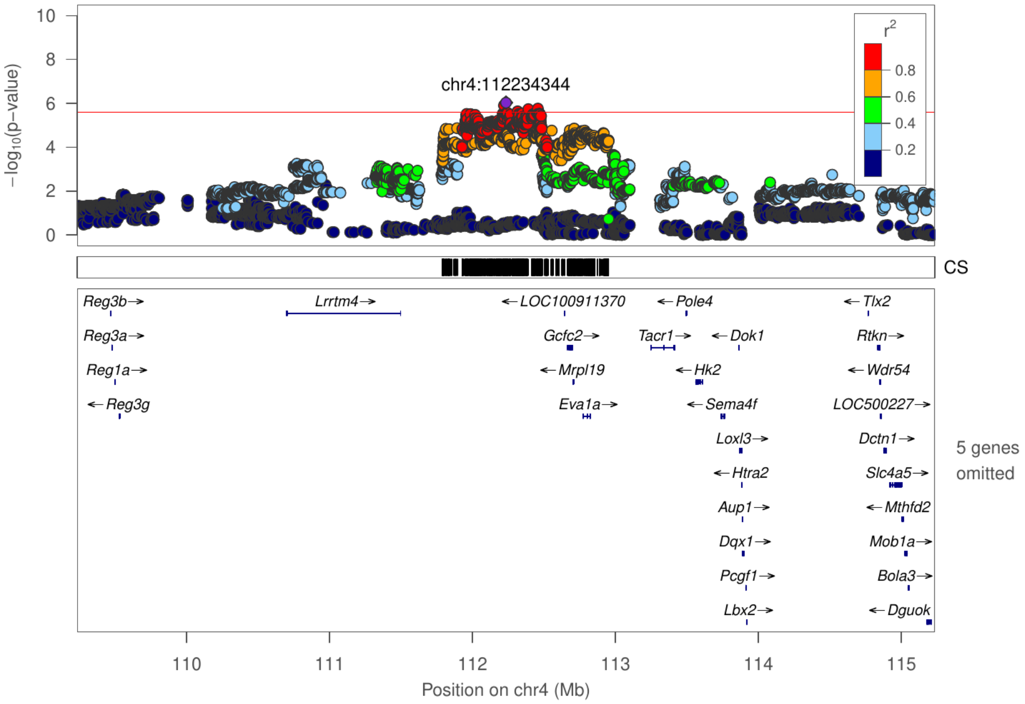

Figure S32. Regional association plot for NOIT: Duration in center zone at chr4:112234344

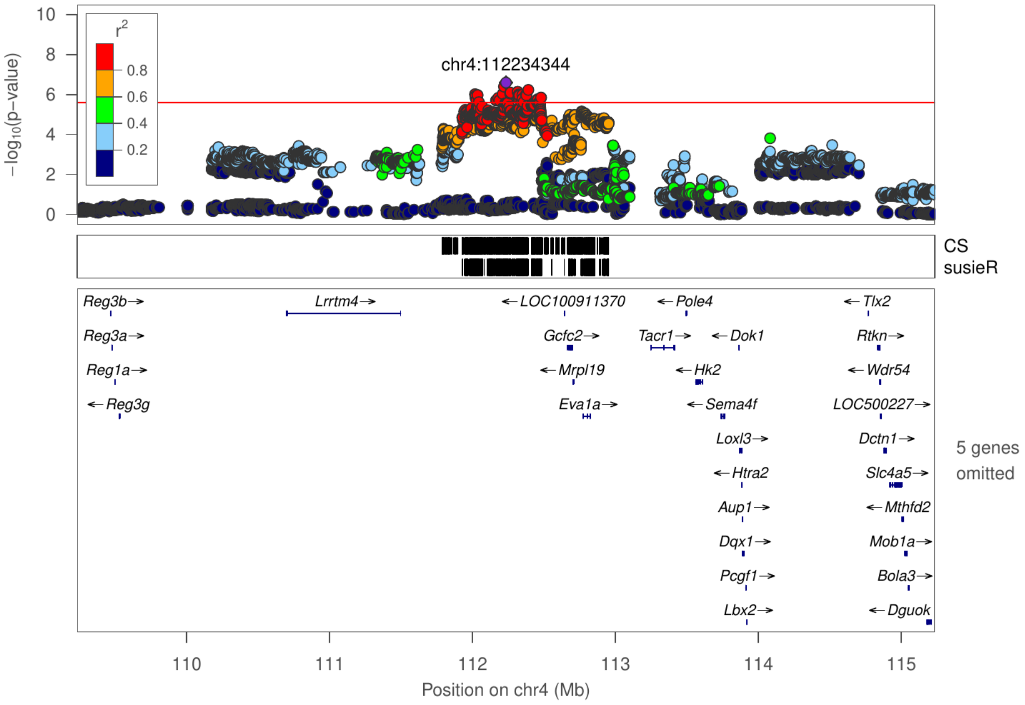

Figure S33. Regional association plot for NOIT: Mean distance to center zone at chr4:112234344

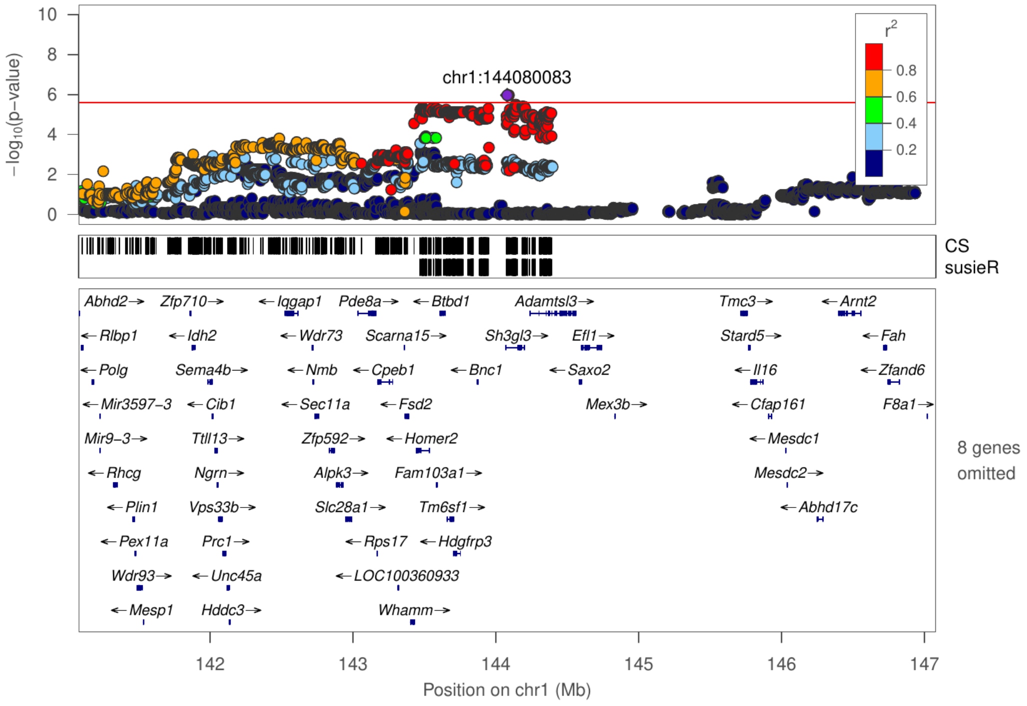

Figure S34. Regional association plot for NOIT: Total distance to center zone at chr1:144080083

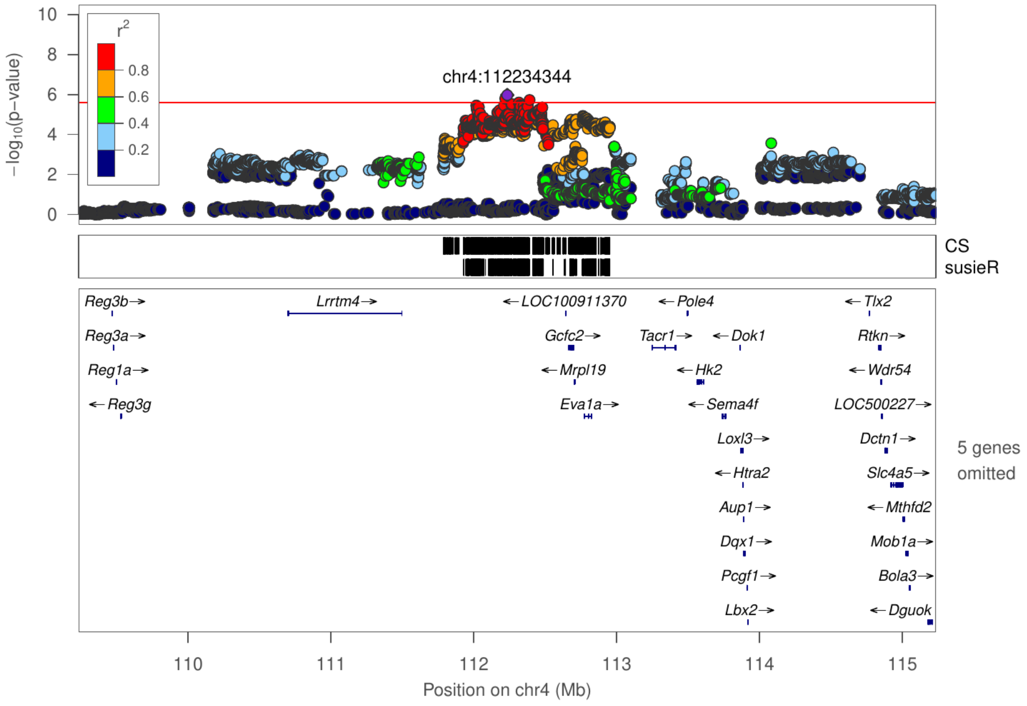

Figure S35. Regional association plot for NOIT: Total distance to center zone at chr4:112234344

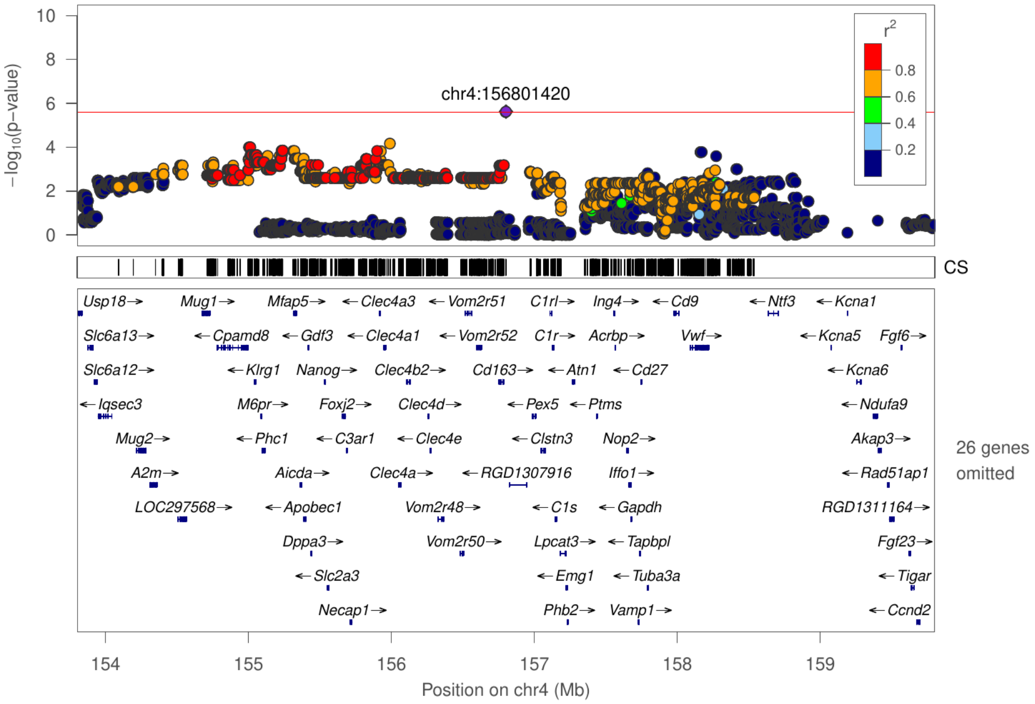

Figure S36. Regional association plot for NOIT: Total distance to center zone at chr4:156801420

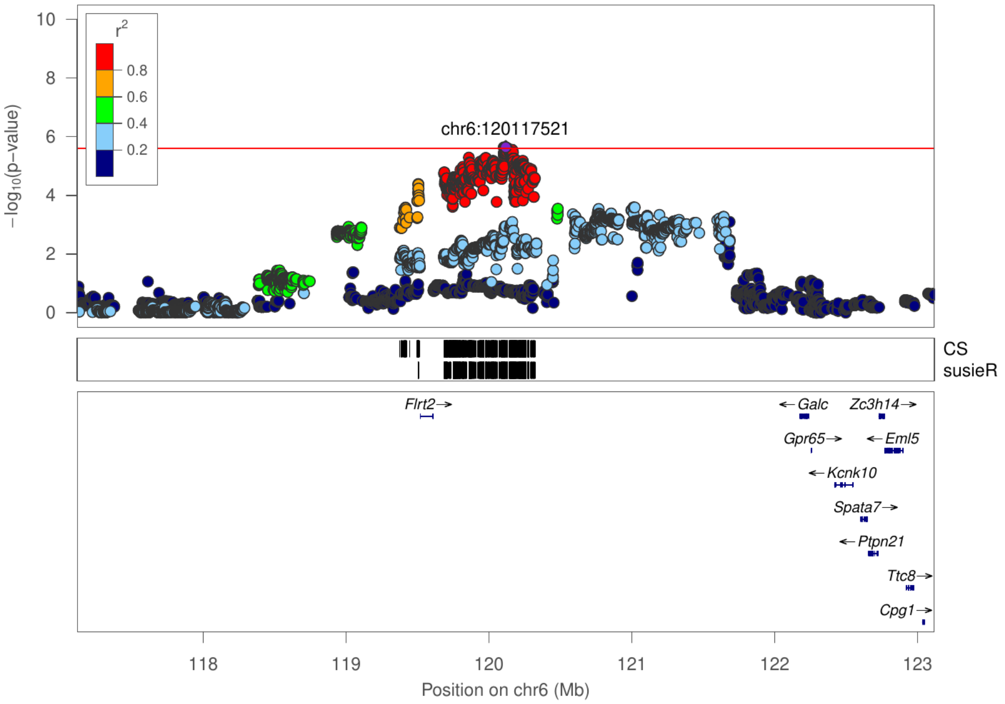

Figure S37. Regional association plot for NOIT: Total travel distance at chr6:120117521

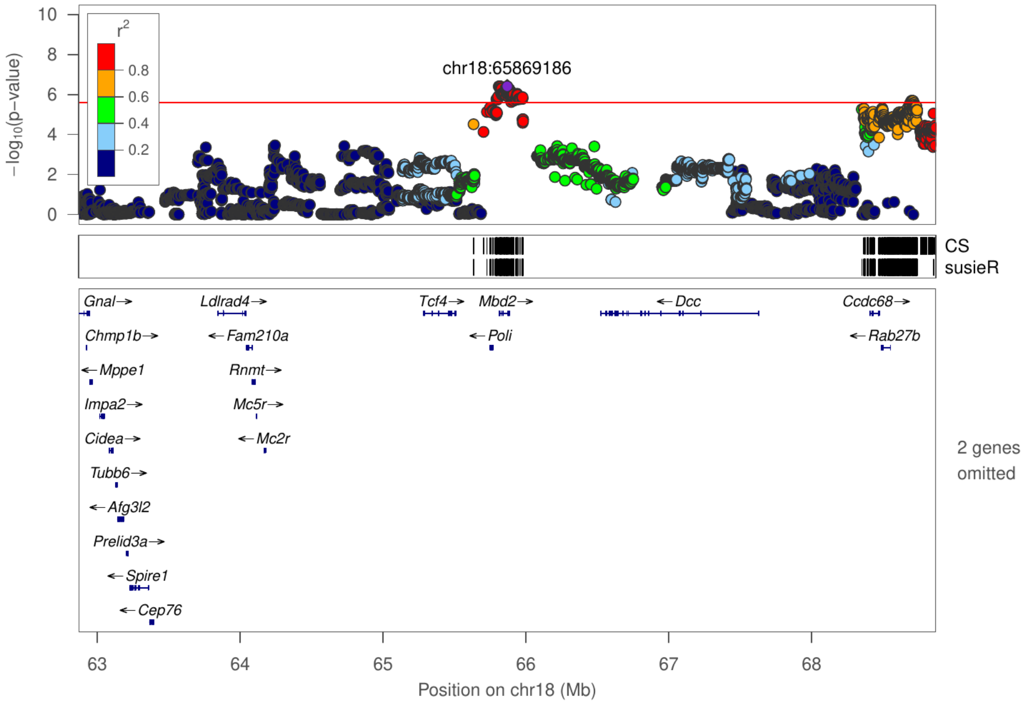

Figure S38. Regional association plot for SIT: Duration in object zone at chr18:65869186

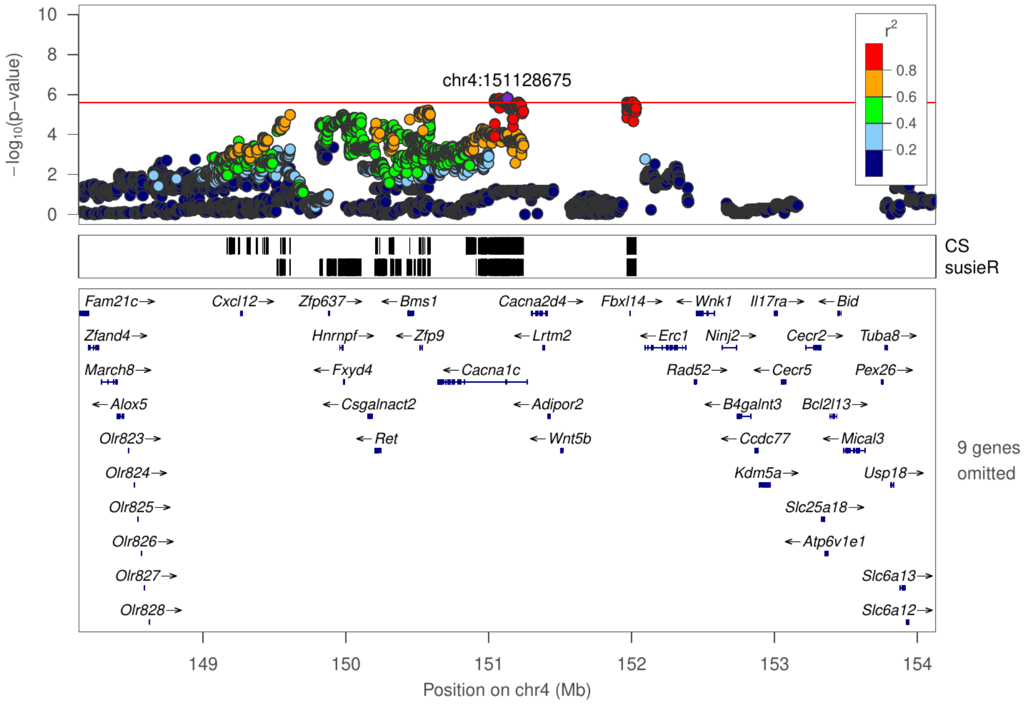

Figure S39. Regional association plot for SIT: Duration in social zone at chr4:151128675

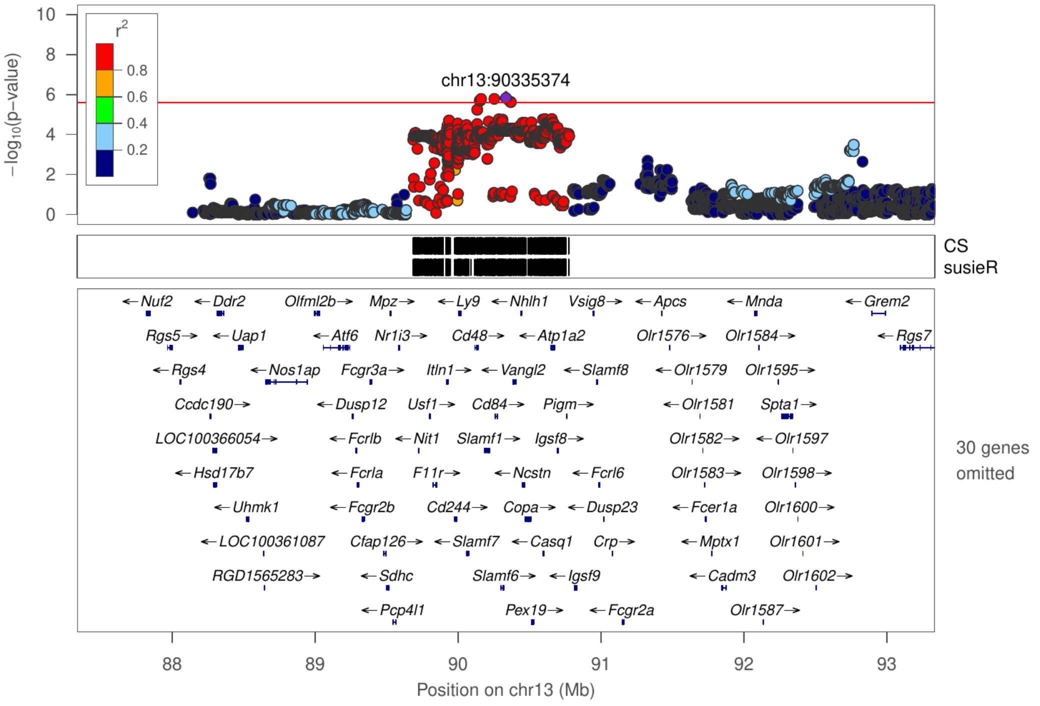

Figure S40. Regional association plot for SIT: Frequency of entering object zone at chr13:90335374

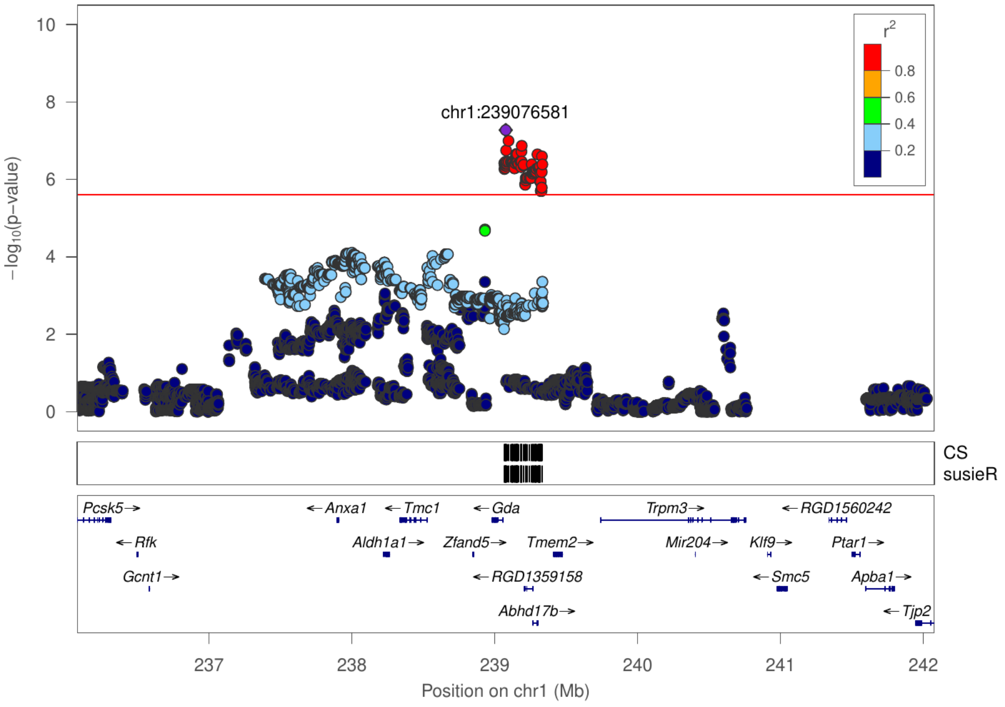

Figure S41. Regional association plot for SIT: Frequency of entering social zone at chr1:239076581

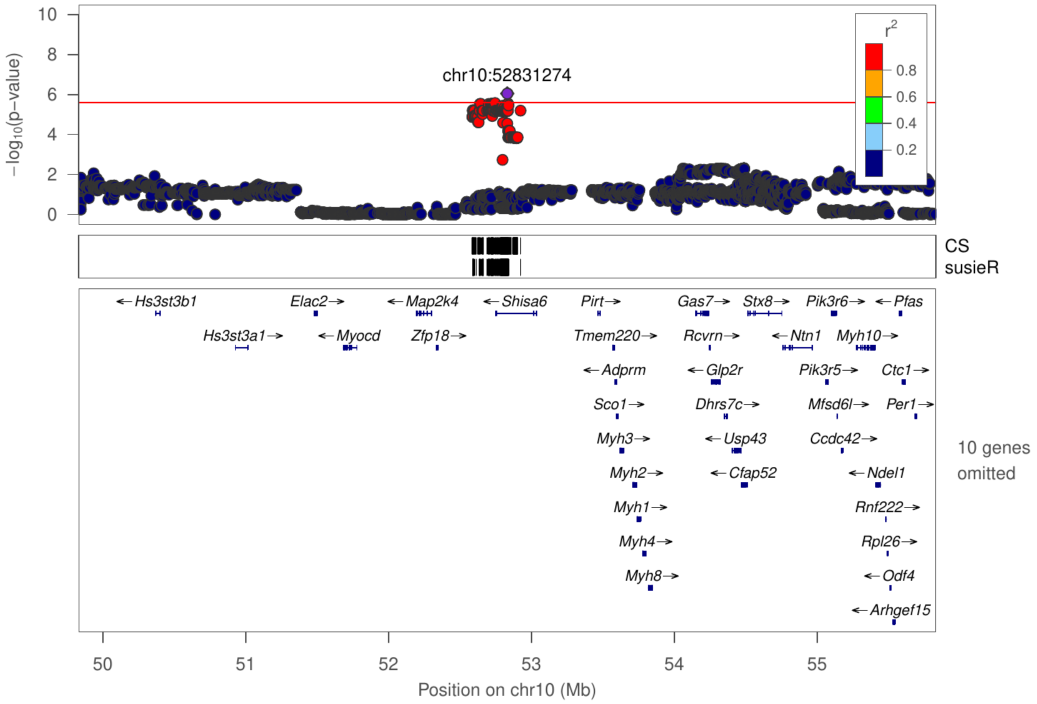

Figure S42. Regional association plot for SIT: Latency of entering social zone at chr10:52831274

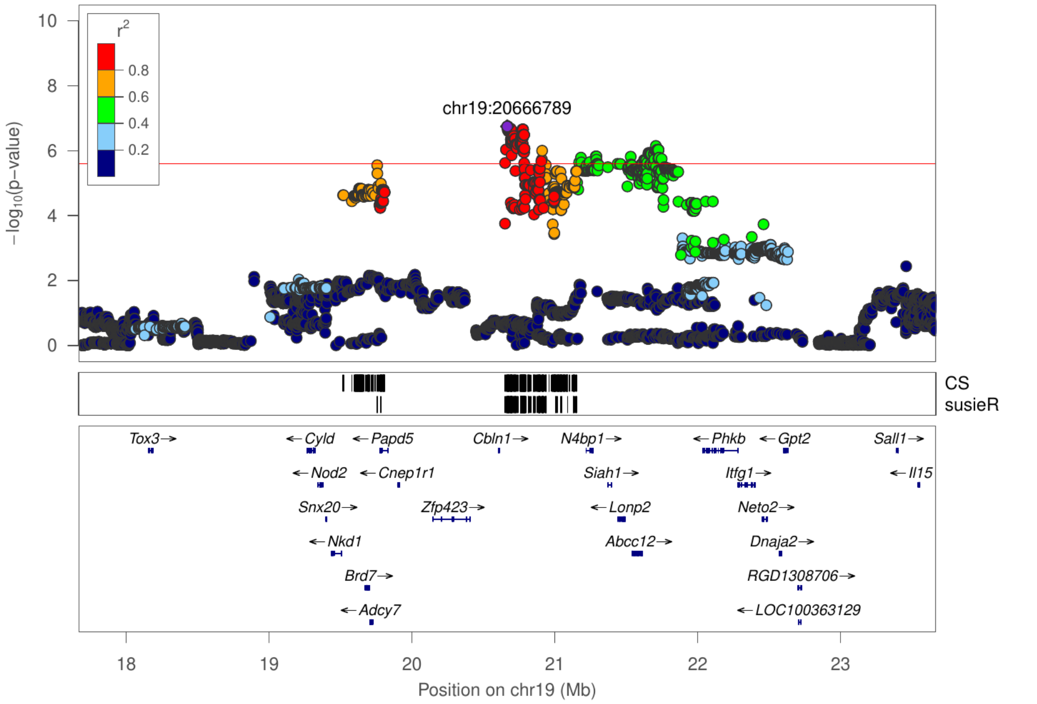

Figure S43. Regional association plot for SIT: Mean distance to object zone at chr19:20666789

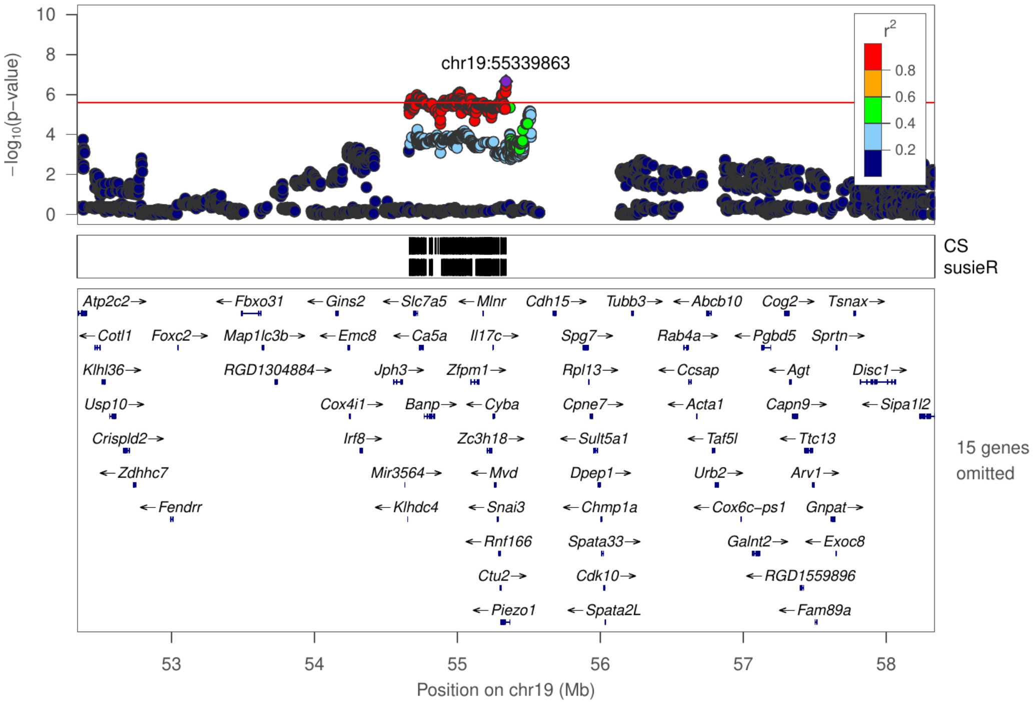

Figure S44. Regional association plot for SIT: Mean distance to social zone at chr19:55339863

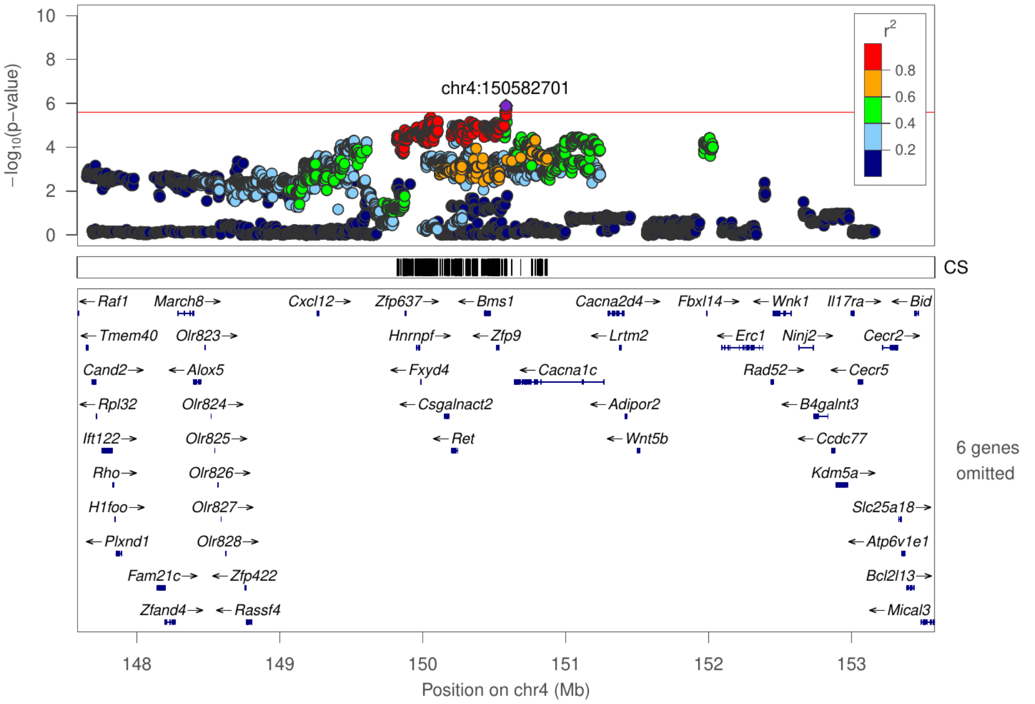

Figure S45. Regional association plot for SIT: Mean distance to social zone at chr4:150582701

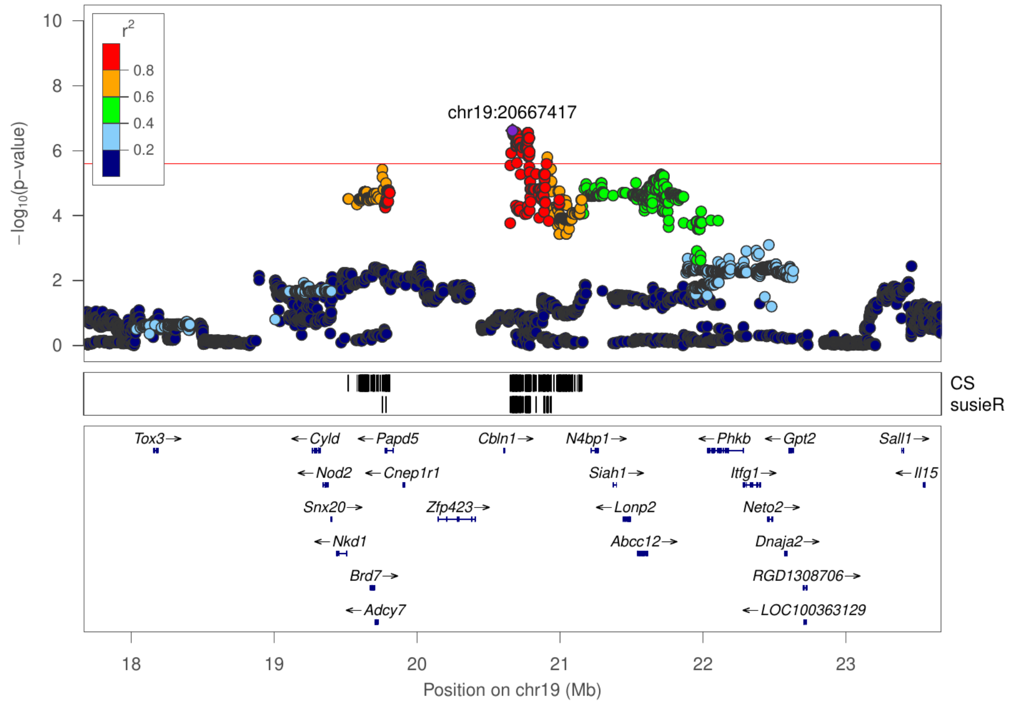

Figure S46. Regional association plot for SIT: Total distance to object zone at chr19:20667417

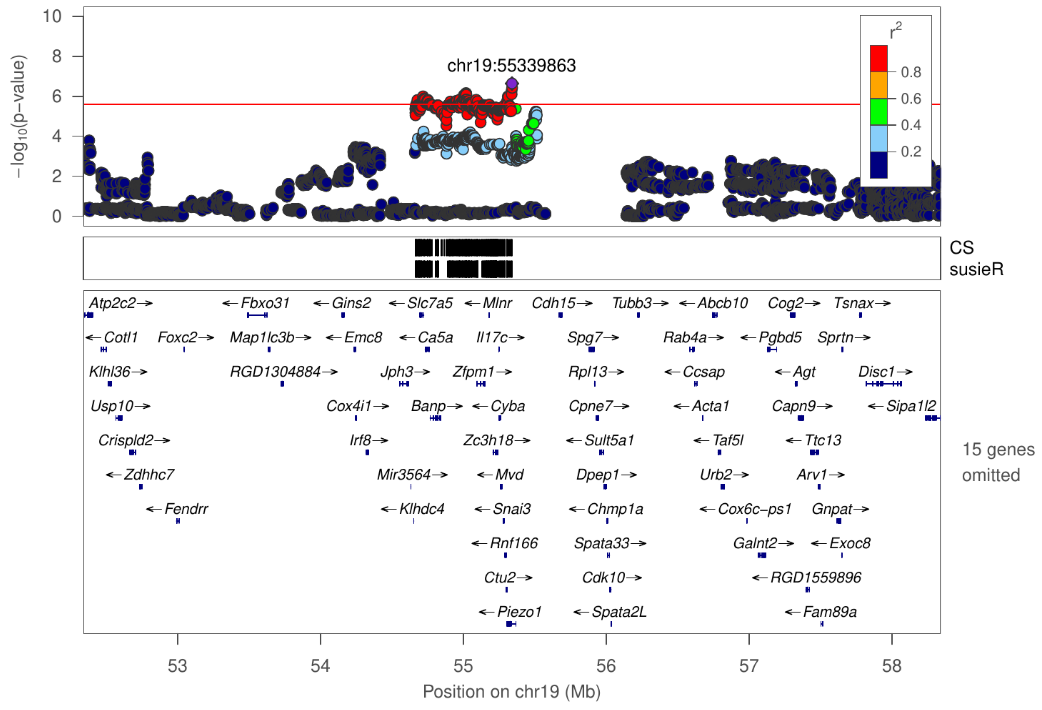

Figure S47. Regional association plot for SIT: Total distance to social zone at chr19:55339863

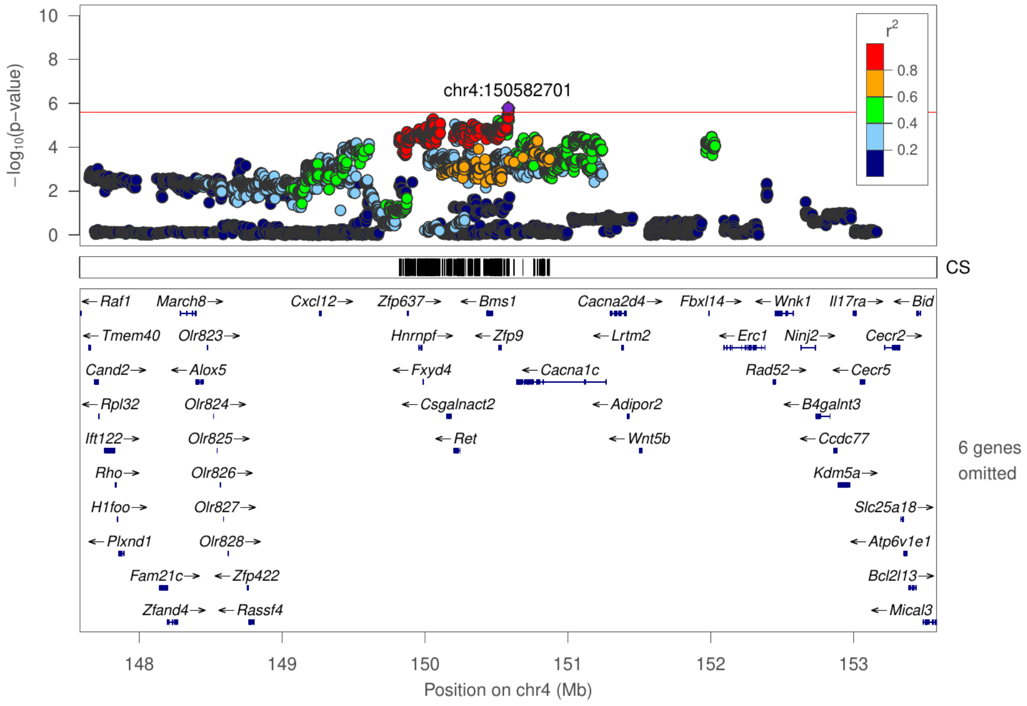

Figure S48. Regional association plot for SIT: Total distance to social zone at chr4:150582701

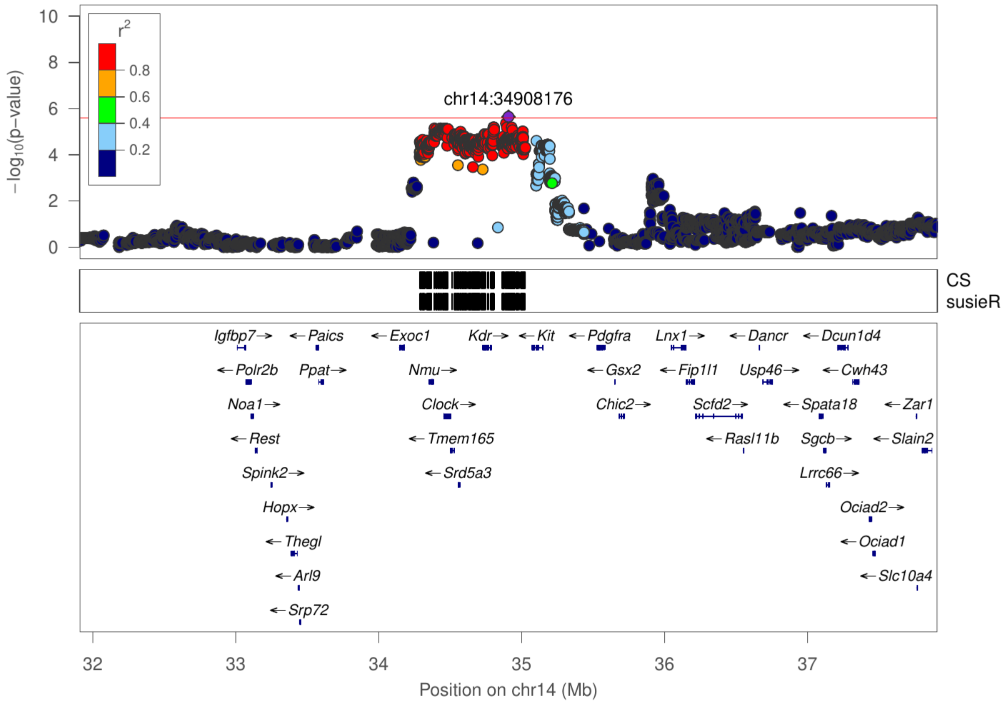

Figure S49. Regional association plot for SIT: Total travel distance at chr14:34908176

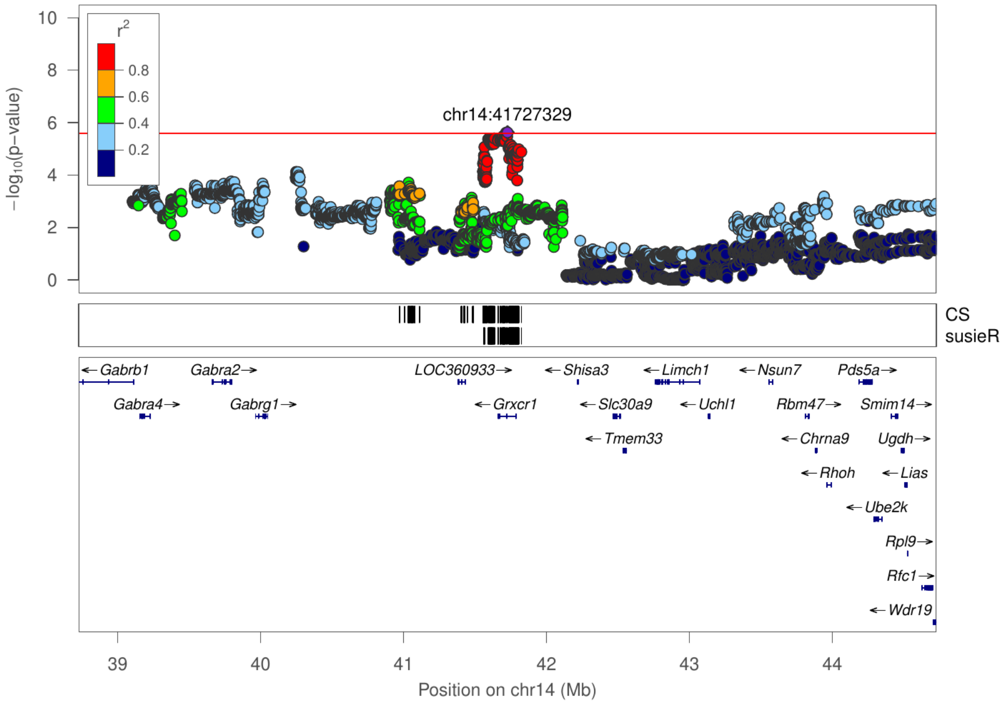

Figure S50. Regional association plot for SIT: Total travel distance at chr14:41727329
